# Implications of Coastal Conditions and Sea‐Level Rise on Mangrove Vulnerability: A Bio‐Morphodynamic Modeling Study

**DOI:** 10.1029/2021JF006301

**Published:** 2022-02-28

**Authors:** Danghan Xie, Christian Schwarz, Maarten G. Kleinhans, Zeng Zhou, Barend van Maanen

**Affiliations:** ^1^ Faculty of Geosciences Utrecht University Utrecht Netherlands; ^2^ Department of Civil Engineering Hydraulics and Geotechnics KU Leuven Leuven Belgium; ^3^ Department of Earth and Environmental Sciences KU Leuven Leuven Belgium; ^4^ State Key Laboratory of Hydrology‐Water Resources and Hydraulic Engineering Hohai University Nanjing China; ^5^ College of Life and Environmental Sciences University of Exeter Exeter UK

**Keywords:** mangroves, sea‐level rise, bio‐morphodynamic feedbacks, coastal conditions, accommodation space, numerical modeling

## Abstract

Mangrove forests are valuable coastal ecosystems that have been shown to persist on muddy intertidal flats through bio‐morphodynamic feedbacks. However, the role of coastal conditions on mangrove behavior remains uncertain. This study conducts numerical experiments to systematically explore the effects of tidal range, small wind waves, sediment supply and coastal slope on mangrove development under sea‐level rise (SLR). Our results show that mangroves in micro‐tidal conditions are more vulnerable because of the gentler coastal equilibrium slope and the limited ability to capture sediment, which leads to substantial mangrove landward displacement even under slow SLR. Macro‐tidal conditions with large sediment supply promote accretion along the profile and platform formation, reducing mangrove vulnerability for slow and medium SLR, but still cause rapid mangrove retreat under fast SLR. Small wind waves promote sediment accretion, and exert an extra bed shear stress that confines the mangrove forest to higher elevations with more favorable inundation regimes, offsetting SLR impacts. These processes also have important implications for the development of new landward habitats under SLR. In particular, our experiments show that landward habitat can be created even with limited sediment supply and thus without complete infilling of the available accommodation space. Nevertheless, new accommodation space may be filled over time with sediment originating from erosion of the lower coastal profile. Consistent with field data, model simulations indicate that sediment accretion within the forest can accelerate under SLR, but the timing and magnitude of accretion depend non‐linearly on coastal conditions and distance from the mangrove seaward edge.

## Key Points

- Mangroves in micro‐tidal systems are most vulnerable to sea‐level rise (SLR) even under excess sediment supply
- Mangrove seaward edge can stay stable even with fast SLR due to the buffer space created during vegetation recruitment
- Spatial variations in accretion across mangrove forests are non‐linearly influenced by rising sea levels and coastal conditions

## 1 Introduction

Mangrove forests inhabiting the margin between land and sea at low latitudes provide crucial ecosystem services to coastal communities including resource provisioning (e.g., timber and fuelwood), coastal protection, organism habitat (e.g., shrimp and fish) and cultural services (Alongi & Mukhopadhyay, 2015; Barbier et al., 2011; Brander et al., 2012). When submerged by tides, mangrove trees dissipate wave energy and reduce tidal currents, protecting the coast from erosion and hydraulic scouring (Temmerman et al., 2013). Reduced hydrodynamic forces within the forest moreover facilitate settling of suspended sediment, which increases bed elevation and thus promotes wetland survival with rising sea levels (Krauss et al., 2014). Sea‐level rise (SLR) is a major stressor for mangrove forests which are only able to tolerate limited durations of tidal inundation (Woodroffe et al., 2016). Changes in inundation time and depth are not only a function of the rate of SLR, but also dependent on coastal slope and morphological adaptation, which in turn are linked to hydro‐sedimentary boundary conditions such as tidal range, wave action and sediment supply (Blasco et al., 1996; FitzGerald et al., 2008; Passeri et al., 2015). Although field data have revealed that ecosystem services provided by mangroves are non‐linearly related to mangrove coverage (Barbier et al., 2008), the fate of mangrove forests in response to SLR remains debated (Krauss et al., 2014; Woodroffe et al., 2016). Considering the complex interplay between physical processes and vegetation dynamics in such environments (Xie et al., 2020), it is still unclear how non‐linear bio‐physical feedbacks determine mangrove survival under varying boundary conditions. As such, in order to develop accurate vulnerability assessments of mangrove forests, we need to systematically evaluate mangrove behavior and their potential future responses to SLR under a broad range of coastal conditions (Friess et al., 2019).

The balance between SLR and sediment accretion is often suggested to be the controlling factor in determining mangrove vulnerability under SLR (Lovelock et al., 2015). Regions with fast SLR and low sediment availability are shown to experience increased flooding leading to tree mortality at seaward mangrove margins and landward migration in the absence of any obstructions (Gilman et al., 2008). In contrast, mangrove forests located in regions with high sediment availability are shown to expand seaward despite rising sea levels (de Jong et al., 2021; Nardin et al., 2016). Although mangroves can elevate the bed through organic accumulation in addition to trapping suspended sediment (Krauss et al., 2014), the net surface elevation change is additionally governed by other sub‐surface processes (shallow subsidence, remineralization and compaction of organic sediment) which may offset this elevation gain (McKee, 2011; McKee et al., 2021). In wetland models, the availability of suspended sediment is typically assumed to be constant across the wetland surface or dependent on distance from the wetland edge (Kirwan & Murray, 2007; Kirwan et al., 2010; Mogensen & Rogers, 2018; Schuerch et al., 2018; Swanson et al., 2014; Thorne et al., 2018), implying that wetland accretion will accelerate under SLR‐driven increases in inundation. However, recent mangrove modeling has shown that such an assumption can lead to an underestimation of bio‐physical effects inherent to the complex interactions between vegetation dynamics and hydro‐sedimentary processes (Xie et al., 2020). Both vegetation presence and bio‐physical effects are ultimately controlled by the coastal conditions, including hydrodynamic forcing and sediment supply (Chapman, 1976; Woodroffe et al., 2016). Thus, investigating mangrove survival under a range of environmental factors becomes imperative.

**Figure 1:**
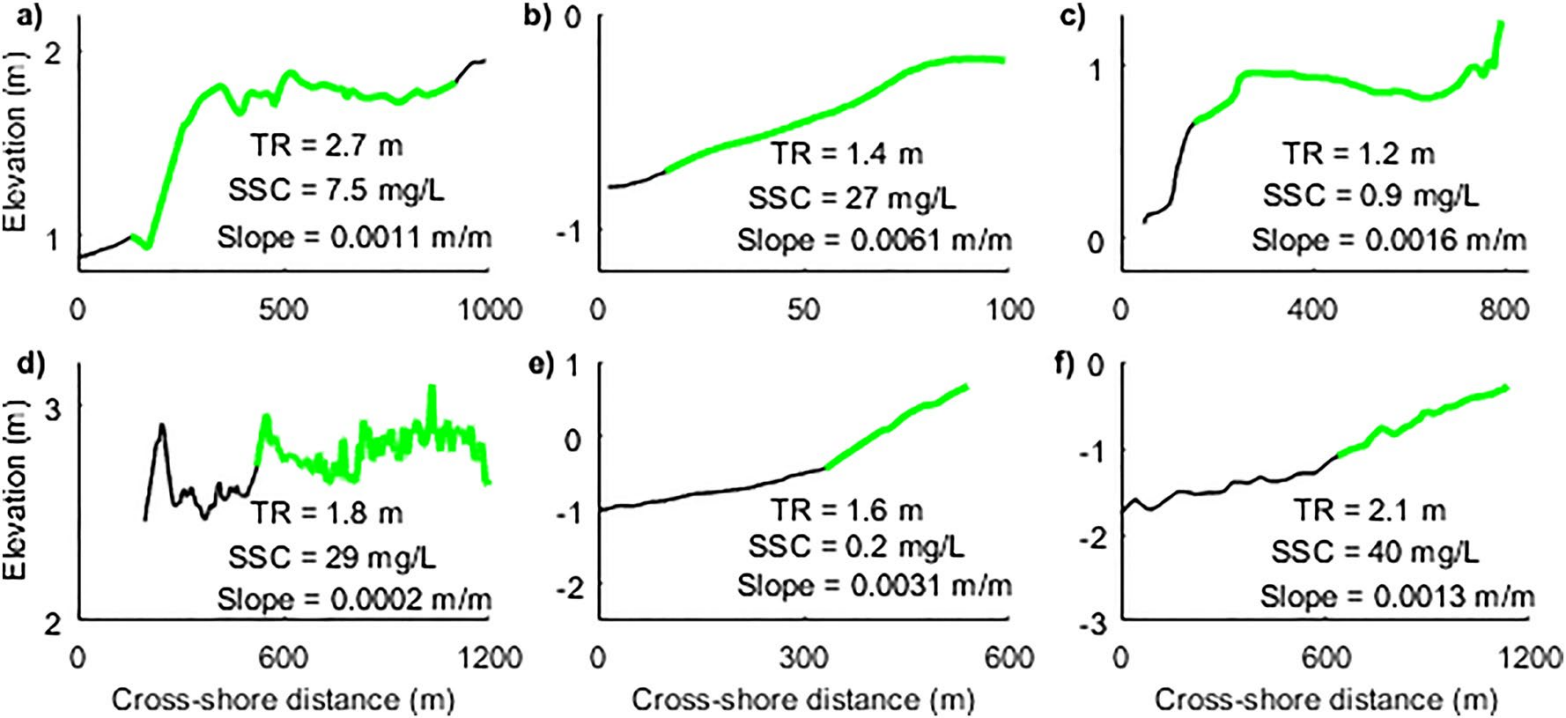
Cross‐shore profiles of mangrove environments adapted from Bryan et al. (2017). (a): Firth of Thames, New Zealand (Swales et al., 2007); (b) Ba Lat Estuary, Red River Delta, Vietnam (Van Santen et al., 2007); (c) Glasshouse Mountains Creek, Queensland, Australia (tidal creek occurs further seaward) (Knight et al., 2009); (d) Macouria mud bank, French Guiana (Proisy et al., 2009); (e) Kantang Estuary in Thailand (Horstman et al., 2014); and (f) Cù Lao Dung in the Mekong River Delta, Vietnam (Bryan et al., 2017). Green dots indicate mangrove presence. TR is annual mean tidal range calculated from TPXO tide models (https://www.tpxo.net/home) and SSC is annual mean suspended sediment concentration derived from the satellite‐borne GlobColor data (http://globcolor.info). Slope is based on the two endpoints of the profile. Note that these cross‐shore profiles and vegetation distributions may not fully cover the landward portion of the forest, meaning the natural profile slopes could be different from slopes shown above.

Mangroves are found in intertidal areas with different coastal slopes and varying profile shapes (Figure 1), which have a governing effect on the response of mangroves to rising sea levels. Previous research has shown that the development of bare coastal profiles is significantly determined by the coastal conditions. Under dominant tidal forcing, coastal profiles develop a more convex equilibrium shape, whereas the addition of waves favors a more concave equilibrium shape (Friedrichs, 2011; Waeles et al., 2004). Furthermore, the balance between tidal range and sediment supply plays a significant role in determining profile slopes. Previous numerical simulations indicate that the equilibrium bare slope becomes steeper/flatter with a higher/lower tidal range and lower/higher sediment supply (Roberts et al., 2000). Coastal profiles in nature do not typically remain bare, but rather often get colonized by vegetation to become intertidal wetlands situated around the mean high water mark (Cahoon et al., 2020). Enhanced drag caused by the presence of mangrove forests contributes to the development of a more convex profile, compared with non‐vegetated or sparse‐vegetated flats, which may create a more linear profile (Bryan et al., 2017). Since the coastal profile controls water‐ and sediment transport and derived parameters such as flooding depth and duration, it consequently controls habitat suitability for mangroves under SLR (Krauss et al., 2014). Moreover, tidal range and the associated strength of tidal currents also determine the extent of sediment transport to higher intertidal areas, and thus sediment accumulation and development of the vegetated platform (Y. Chen et al., 2018). Previous research also suggests that SLR will cause a greater relocation of mangroves in micro‐tidal areas relative to macro‐tidal areas assuming similar coastal slopes (Ellison, 2015). Thus, coastal slope and shape determined by tidal range potentially exert a major impact on mangrove drowning under SLR. For example, the drowning of a coastal plateau means a sudden set‐back of mangrove vegetation, emphasizing the need to study the interaction between tidal dynamics, sediment transport, coastal slope and how this influences mangrove survival.

In addition to tidal currents, wind waves exert additional bed shear stress enhancing sediment resuspension (Green & Coco, 2014) while mangroves protect the coast through dampening wave energy and facilitating net sediment deposition (Horstman et al., 2014; Mazda et al., 1997). Recent research highlights the importance of small wind waves in determining the long‐term stability of salt marsh systems (Schuerch et al., 2019). However, for mangrove sites, the majority of existing studies have focused on the dissipation of energetic waves by vegetation (Bao, 2011; Horstman et al., 2014; Mazda et al., 1997). The effects of small but frequently occurring wind waves on morphological evolution in mangrove ecosystems have received less attention (Green, 2011; Horstman et al., 2014; Van Santen et al., 2007). On the one hand, small waves (with significant wave height less than 20 cm) are capable of resuspending sediment over tidal flats (Green, 2011; Green et al., 1997; Uncles & Stephens, 2000) thereby potentially increasing sediment availability to adjacent wetlands and compensating the sediment deficit in sediment accumulation (Carling, 1982). On the other hand, shifting the limiting factors from sediment availability to sediment transport capacity may be altered by mangroves, causing spatial variations in accumulation rate (Swales et al., 2019). From an ecological point of view, strong tidal currents and waves limit the establishment of mangrove seedlings due to increased shear stresses so that the seaward edge of mangrove forests is not always at mid‐tidal elevation (Balke et al., 2011; Friess et al., 2012). Such colonization restrictions not only control initial mangrove coverage, but potentially also affect mangrove responses under rising sea levels.

To understand and project future coastal development, we need to identify processes driving mangrove vulnerability under different coastal conditions. Global and regional assessments on mangrove vulnerability using first‐order approximations show how increased vertical accretion can mitigate the effects of rising sea levels (Lovelock et al., 2015; Schuerch et al., 2018). Other field site‐specific approaches predict morphological evolution and spatial mangrove development utilizing sophisticated interactions between mangroves and hydrodynamics but simplify non‐linearities in sedimentary processes or exclude dynamic feedbacks between mangrove growth and coastal geomorphological change (Bryan et al., 2017; Rodriguez et al., 2017). Other more recent modeling studies have incorporated a broader range of dynamic feedbacks between mangrove growth and coastal geomorphological change (Breda et al., 2021), highlighting the potential and need to use such bio‐morphodynamic models to assess mangrove resilience in various coastal conditions. Currently, different views exist on the fate of coastal wetlands, with some studies indicating that wetland area can increase as long as space for landward migration is available (Schuerch et al., 2018), while others suggest that longer‐term wetland loss is inevitable (Törnqvist et al., 2021). Addressing this dispute requires new modeling approaches that account for dynamic vegetation growth (i.e., colonization, growth and mortality processes) and comprehensive treatment of hydro‐sedimentary processes, including the development of newly generated accommodation space created by SLR which can potentially be filled in with sediment. Such modeling efforts have to be able to explore mangrove dynamics under varying boundary conditions to unravel the broad range of potential mangrove responses. In this study, we fill this knowledge gap and utilize a bio‐morphodynamic numerical model to determine how non‐linear bio‐physical interactions control coastal profile evolution and mangrove coverage in the face of SLR in various coastal conditions. This will help to elucidate the key controlling factors governing mangrove vulnerability. To this end, we conducted 72 simulations to systematically explore the effects of tidal range, wave action, sediment supply and coastal slope under three different SLR scenarios.

## 2 Methods

Mangrove behaviors are simulated by considering different combinations of coastal conditions, including SLR, tidal range, wind waves (hereafter referred to as waves), sediment supply and coastal slope. Here we use a recently developed bio‐morphodynamic model, which considers a two‐way interaction between physical and biological processes (Xie et al., 2020). The model couples an open‐source hydro‐morphodynamic model (i.e., Delft3D, version 4.01.00) containing flow, wave and sediment transport modules (Lesser et al., 2004), and a dynamic vegetation model modified after van Maanen et al. (2015). The hydro‐morphodynamic model provides information on local hydroperiod and bed shear stress to the vegetation model where this influences vegetation dynamics. The vegetation model in turn feeds the hydro‐morphodynamic model with updated vegetation information so that vegetation‐induced flow resistance and bed roughness can be accounted for. Although mangroves have the capacity to elevate their soils through organic matter accretion, enhancing their ability to keep pace with SLR (Krauss et al., 2014; Woodroffe et al., 2016), other sub‐surface processes such as subsidence due to decomposition and sediment auto‐compaction may decrease elevation, increasing the risk of mangroves being submerged (Rogers et al., 2005). We therefore decided to exclude these counteracting processes and focus on above‐ground bio‐morphodynamic feedbacks. Considering the number of scenarios that need to be tested, a one‐dimensional cross‐shore profile model is constructed, assuming alongshore uniformity in morphology and boundary conditions in order to reduce model complexity and computational time similar to previous studies (Roberts et al., 2000; Xie et al., 2020; Zhou et al., 2016). As the model does not incorporate the specific fluvial or estuarine characteristics affecting mangrove behaviors, our modeling study is set up to represent mangrove systems located in open coast systems, broad embayments or tidal dominated deltaic environments, such as the Suriname coast (de Jong et al., 2021), Firth of Thames (Swales et al., 2007) and the Mekong Delta (Nardin et al., 2016). Still, the boundary and initial conditions are simplified in order to keep model output as transparent as possible and unravel the key feedbacks leading to system responses (Murray, 2013; van der Wegen et al., 2011).

### 2.1 Hydro‐Morphodynamic Model

The Delft3D model suite, which has been widely used in both engineering applications and scientific research, is adopted to process hydro‐morphodynamic calculations. Since the parameterization of the hydro‐morphodynamic model is similar to the model in previous research (Xie et al., 2020), here it will only be briefly discussed. The Delft3D model computes flow fields, sediment transport and related profile changes. Water level and flow velocity are calculated by solving the shallow water equations and then are utilized to compute sediment transport. Mangroves are commonly found in muddy environments (Woodroffe et al., 2016), which can exhibit a range of cohesive behavior with varying critical erosion thresholds and erosion rate parameters determined by their sediment composition (van Ledden et al., 2004). In this research, we simplify the effects of different sediment fractions by only considering pure cohesive muddy sediment which can be eroded or deposited over the profile. The erosion and deposition fluxes of mud are calculated through the Partheniades‐Krone formulations (Partheniades, 1965) and sediment is transported according to the advection‐diffusion equation. The critical shear stress for erosion is set to 0.2 N/m^2^ and the threshold for sedimentation is set to 1,000 N/m^2^, following previous studies (Xie et al., 2020; Zhou et al., 2016). Other hydro‐morphodynamic parameter settings can be found in Table S1 of Supporting Information S1. Bed level change will then be updated considering net changes in sediment fluxes following the principle of mass conservation and fed back instantly to update the flow field in order to start the next hydrodynamic calculation.

We applied the “roller model” option to mimic wave action across the 1‐D profile following van der Wegen et al. (2017). The roller model includes the effects of short waves on long waves. The application of the roller model is appropriate for short waves where the wave spectrum is narrow‐banded for both frequency and direction. The roller energy is transformed from wave energy through wave breaking and is rapidly dissipated in shallow regions (Nairn et al., 1991; Reniers et al., 2004; Tajziehchi, 2006). Propagation of waves and roller energy associated with the dissipation of wave energy will cause spatiotemporal variations in wave characteristics and roller energy and thus a variation in radiation stress, which will influence bed shear stress resulting in extra sediment mobility (Reniers et al., 2002, 2004). We applied an online coupling between the roller wave model and the flow model to account for wave‐current interactions. In contrast to the strong interactions between large‐wave energy and mangrove behaviors, we here focus on the effects of small waves that may control morphological change and related mangrove vulnerability over longer time scales, but which have so far received relatively little attention. As such, we apply a small wave height (i.e., significant wave height = 5 cm) on the seaward boundary. Although the flow is affected by vegetation, the effects of mangroves on wave damping are not included in the current model framework. Therefore, our results may overestimate the role of waves in morphological evolution.

### 2.2 Dynamic Vegetation Model

The behaviors of mangrove forests are modeled in Matlab (R2017a), including the processes of colonization, growth and mortality, after van Maanen et al. (2015). For clarity, the key concepts of the vegetation model are described in detail. To realize the interaction between vegetation dynamics and hydro‐morphodynamic processes, we couple the vegetation model with the hydro‐morphodynamic model (Delft3D) described above, based on the framework introduced in previous studies (Brückner et al., 2019; van Oorschot et al., 2016). Here, the physical conditions determine vegetation colonization, growth and mortality, while the vegetation properties determine hydraulic resistance and bed roughness.

To account for vegetation effects in the hydro‐morphodynamic model, we applied the trachytope approach with the Baptist formula (Baptist et al., 2007) that allows for multiple fractions of different vegetation characteristics existing within one numerical cell, including both stems and roots. A net roughness *C*_*n*_ (m^1/2^/s) and additional resistance term *M* in both *x* and *y* directions (i.e. $M_{x}=-\frac{\lambda }{2}u^{2}\;and\;M_{y}=-\frac{\lambda }{2}v^{2}$) are incorporated in the hydro‐morphodynamic model in the presence of mangroves. *u* and *v* are the depth‐averaged flow velocity in *x* and *y* directions, respectively. Both *C*_*n*_ and *λ* are calculated from vegetation characteristics and water depth (*h*; unit: m):

(1) $$C_{n}=\left\{\begin{array}{ll} C_{b}+\frac{\sqrt{g}}{\kappa }\ln\left(\frac{h}{h_{v}}\right)\sqrt{1+\frac{C_{D}nh_{v}C_{b}^{2}}{2g}} & ,\text{if}\;h\geq h_{v}\\ C_{b} & ,\text{if}\;h<h_{v} \end{array}\right.$$

(2) $$\lambda =\left\{\begin{array}{ll} C_{D}n\frac{h_{v}}{h}\frac{C_{b}^{2}}{C_{n}^{2}} & ,\text{if}\;h\geq h_{v}\\ C_{D}n & ,\text{if}\;h<h_{v} \end{array}\right.$$

where *C*_*b*_ is the non‐vegetated Chézy coefficient, set to 65 m^1/2^/s; *g* = 9.81 m/s^2^ is the gravity; *κ* = 0.41 is the Von Kármán constant; *h*_*v*_ (m) is the height of vegetation objects (stems or roots); *C*_*D*_ (−) is the drag coefficient accounting for roots and stems of mangroves, which can vary greatly between 0.7 and 3.5 (Horstman et al., 2018; Nepf, 1999). Here we set *C*_*D*_ of tree stems to 1.5 and *C*_*D*_ of roots to 1 following previous studies (van Oorschot et al., 2017; Xie et al., 2020). The sensitivity analysis of *C*_*D*_ can be seen in Figure S1 of Supporting Information S1. *n = mD* where *m* is the number of vegetation objects per square meter and *D* is the diameter of these objects. When different sizes of vegetation objects co‐exist in one numerical cell, *C*_*n*_ and *λ* are calculated separately for each vegetation object and averaged with the specific fraction coverage of each vegetation object.

The ecological time step is set to 1 month so that the status of mangrove trees is updated 12 times per year. At the end of every ecological time step, the vegetation model requests result from the hydro‐morphodynamic model, such as bed levels, water levels and bed shear stresses, to update vegetation characteristics, including tree diameter, height, density and their corresponding root elements, serving as new input for the next hydro‐morphodynamic simulation. The seedlings colonize at the beginning of the year (i.e., 1st ecological time step), and at the end of the year (i.e., 12th ecological time step) the growth quality of each mangrove is evaluated as a basis for mangrove mortality.

*Avicennia marina* has been chosen as a representative mangrove species following a previous mangrove modeling study (van Maanen et al., 2015). Although mangrove growth patterns can be more complex (Kumbier et al., 2021), the habitat of *Avicennia marina* has been previously approximated as the area between mean sea level and mean high water level (Clarke & Myerscough, 1993). In the model, the hydrodynamic conditions for mangrove seedling establishment are (a) appropriate inundation regime and (b) limited hydrodynamic forces, both of which are evaluated at the first ecological time step. Relative hydroperiod, *P*, calculated as the proportion of time that one cell is inundated within one ecological time step, has been adopted to evaluate whether the inundation regime is appropriate for mangrove seedlings to settle. Here, for *Avicennia marina*, we assume grid cells inundated less than half of the time (i.e., 0 < *P* < 0.5) are appropriate for mangrove colonization. Apart from this inundation requirement, limited hydrodynamic force is also required for successful seedling settlement, which has been regarded as windows of opportunity (Balke et al., 2011; Friess et al., 2012). Previous physical experiments show that wave‐ or current‐induced hydrodynamic force determines seedling establishment, for example, bed shear stresses smaller than 0.2 N/m^2^ will allow most seedlings to settle without being uprooted (Balke et al., 2011). Thus, for suitable seedling establishment areas, we calculate the 90th percentile bed shear stress at the end of every year and adopt 0.2 N/m^2^ as a threshold under which mangrove colonization can occur. The initial number of seedlings is set to 30 per 100 m^2^ which is also the maximum density that one grid cell can accommodate and the initial stem diameter is set to 1.37 cm (Berger & Hildenbrandt, 2000; van Maanen et al., 2015).

Mangrove growth in our model refers to the increase of stem diameter (*D*; cm), tree height (*H*; cm) and the number of corresponding root elements. The growth rates are assumed to relate to local flooding and available resources, described by the following relations (Berger & Hildenbrandt, 2000; R. Chen & Twilley, 1998; van Maanen et al., 2015):

(3) $$\begin{array}{l} \frac{dD}{dt}=\frac{GD\left(1-\frac{DH}{D_{\text{max}}H_{\text{max}}}\right)}{\left(274+3b_{2}D-4b_{3}D^{2}\right)}\cdot f\cdot C \end{array}$$

(4) $$\begin{array}{l} H=137+b_{2}D-b_{3}D^{2} \end{array}$$

where *t* is time (month). *D*_max_ and *H*_max_ are maximum stem diameter and tree height, set to 40 and 1,000 cm, respectively. *G*, *b*_2_, and *b*_3_ are growth constants which are set here to, respectively, 12.68 cm/month, 43 and 0.536 cm^−1^ such that a maximum increase in stem diameter is approximately 1 cm per year. *f* is a fitness function which is an inundation‐based factor to define mangrove growth conditions (Figure S2a in Supporting Information S1), calculated as follows (van Maanen et al., 2015):

(5) $$\begin{array}{l} f=a\cdot P^{2}+b\cdot P+c \end{array}$$

where *a*, *b*, and *c* are constants determining the appropriate growth area of mangroves, set to −8, 4 and 0.5, respectively. The fitness function is set up on the premise that there is an optimal relative hydroperiod (i.e., *f* = 1 when *P* = 0.25) for mangroves and the growth quality will decrease when the relative hydroperiod is either larger or smaller than this optimal value. Meanwhile, the tree growth rate is further reduced by limitations in resources, which is introduced as a competition stress factor (*C*) that captures the competition between one specific tree and surrounding mangrove vegetation. The competition stress factor is implemented as a sigmoid function linked to biomass (Figure S2b in Supporting Information S1) with the following formula (van Maanen et al., 2015):

(6) $$\begin{array}{l} C=\frac{1}{1+\exp\left[d\left(W_{0.5}-W\right)\right]} \end{array}$$

where *d* is a constant controlling the decreasing rate of this function, set to −0.0003. *W* is the total biomass (kg) in one grid cell including both aboveground and belowground biomass. *W*_0.5_ is the biomass when *C* = 0.5 which is a critical value in the model as it defines when mangrove competition may result in tree mortality. To set *W*_0.5_, the aboveground (*W*_*a*_) and belowground (*W*_*b*_) biomass of a single *Avicennia marina* are first evaluated through allometric equations (Komiyama et al., 2008):

(7) $$\begin{array}{l} W_{a}=0.308D^{2.11} \end{array}$$

(8) $$\begin{array}{l} W_{b}=1.28D^{1.17} \end{array}$$

We then apply the “zone of influence” approach by defining a circle around each tree to represent the area from where the tree obtains resources (Berger & Hildenbrandt, 2000):

(9) $$\begin{array}{l} R=10\sqrt{D/2} \end{array}$$

where *R* is the circle radius (m) and *D/*2 represents the stem radius (m). We define a critical status without competition as when one grid cell is full of mature mangroves but each mangrove obtains its resources within its own zone of influence, without interacting with other trees. Hence, *W*_0.5_ can be estimated by:

(10) $$\begin{array}{l} W_{0.5}=\frac{A_{\text{cell}}}{{\left(2\cdot R\right)}^{2}}\cdot W_{\text{mature}} \end{array}$$

where *A*_cell_ is the grid cell surface area (m^2^) and *W*_mature_ is the biomass of one mature mangrove (when *D* = 40 cm). As *A*_cell_ = 2,500 m^2^, the application of Equation 10 leads to *W*_*0.5*_ = 2.61 × 10^4^ kg for each grid cell (Figure S2b in Supporting Information S1).

The mortality of mangrove trees is identified when consecutive depressional growth occurs (Berger & Hildenbrandt, 2000). In this study, we use the value of *f* · *C* to represent mangrove growth quality. At the end of every year, the vegetation model evaluates the growth quality of all mangrove trees by calculating *f* · *C* and a mangrove tree dies when the growth rate has been smaller than 50% of the optimal status (i.e., *f* · *C* < 0.5) for 5 consecutive years (van Maanen et al., 2015). Dead trees are removed to reduce local competition stress until growth conditions (*f* · *C*) exceed again 0.5, or when no vegetation is left. Mangroves with smaller diameters are assumed to be more vulnerable to negative growth condition so these trees are removed first when mangroves with multiple stem diameters coexist in one cell. Considering the potential morphological evolution resulting in a concave profile in the upper‐intertidal area, water may stagnate as possibilities for lateral drainage (e.g., through channels) are absent, leading to a shallow but persistent layer of water causing relative hydroperiods to exceed critical inundation thresholds. In this case, mangroves in these areas are set to stay alive without further growth until the shallow water stagnation disappears. This protects vegetation growing in the upper tidal area against prolonged inundation under the current 1D model settings and has no further physical consequences. This implementation does not significantly change morphological evolution based on our sensitivity tests and in nature, mangroves have been shown to persist in such situations (Bryan et al., 2017).

Mangrove root elements provide significant flow resistance and bed friction due to their dense number and pneumatophores (i.e., aerial roots) can grow to a few tens of centimeters high (Liénard et al., 2016; Mazda et al., 2005). To include the effects of these roots, we relate the number of root elements with stem diameter using a sigmoid function (van Maanen et al., 2015) and assume roots keep a fixed size over time. In this case, an increase of stem diameter will lead to a simultaneous increase of root number and thus increase resistance and friction for water movement. The density of root elements recorded in the literature show a broad range, some areas have only a few roots while others may have more than one thousand roots per square meter (Dahdouh‐Guebas et al., 2007). In our model, we assume a mature *Avicennia marina* can have at most 1,000 roots. The number of roots (*N*_roots_) is evaluated with the following formula (van Maanen et al., 2015):

(11) $$\begin{array}{l} N_{\text{roots}}=\frac{1000}{1+\text{exp}\left[k\left(\frac{D_{\text{max}}}{2}-D\right)\right]} \end{array}$$

where *k* is a constant describing the growth rate of the root number with stem diameter (*D*; cm), here set to 0.3. An overview of the vegetation model settings can be found in Table S2 of Supporting Information S1.

### 2.3 Model Set Up

**Figure 2:**
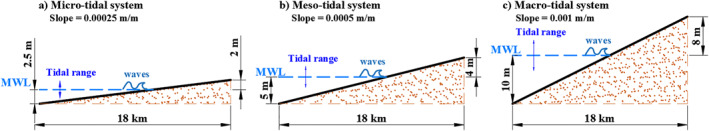
Initial bathymetries with the same cross‐shore distance (18 km) but different bed elevations and slopes. (a) Micro‐tidal system. (b) Meso‐tidal system. (c) Macro‐tidal system. Tidal ranges in micro‐, meso‐ and macro‐tidal systems are set to 1, 3 and 5 m, respectively. The seaward edge bed elevation is −2.5 m in the micro‐tidal system, −5 m in the meso‐tidal system and −10 m in the macro‐tidal system. These settings guarantee that the water depth at the seaward edge is deep enough to accommodate sediment accretion and bed level change even under high sediment supplies. By considering the highest possible elevation that tidal currents can reach under sea‐level rise effects, the landward edge elevation is set to 2 m in the micro‐tidal system, 4 m in the meso‐tidal system and 8 m in the macro‐tidal system.

Mangroves exist in a variety of coastal conditions around the globe. They inhabit tropical/subtropical shorelines characterized by varying tidal ranges, waves, sediment supply and coastal slope. As previously highlighted by Friedrichs (2011), the slope of equilibrium profiles varies with environmental conditions, so based on earlier equilibrium tests (Figure S3 in Supporting Information S1), we create three computational domains with different initial slopes (i.e., gentle, medium, and steep) of 0.00025, 0.0005, and 0.001 m/m to represent different tidal systems (i.e., micro‐, meso‐ and macro‐tidal systems with tidal range of 1, 3, and 5 m; Figure 2). The domain is constructed by 360 grid cells, and each cell size is set to 50 m by 50 m which has shown to be accurate and efficient following previous research (Xie et al., 2020). In a subset, we also include the effects of small wind waves by adding waves with a 5‐cm significant wave height (*H*_*s*_) at the seaward boundary. Peak wave period (*T*_*p*_) is set to 1 s following an approximate empirical relation between *T*_*p*_ and *H*_*s*_ as: $T_{P}\approx 5.3\sqrt{H_{s}}$ (Mangor et al., 2017). Three suspended sediment concentrations (10, 30, and 50 mg/L) at the seaward boundary are applied in our models. Lastly, projections of SLR are implemented by incrementally raising the water level at the seaward boundary following three different representative concentration pathway (RCP) scenarios, from the lowest (5%) quantile of RCP2.6 (29 cm by 2100) through the medium (50%) quantile of RCP4.6 (55 cm by 2100) to the highest (95%) quantile of RCP8.5 (110 cm by 2100; Oppenheimer et al., 2019). A constant morphological acceleration factor (set to 30 based on sensitivity tests) is applied to enable long‐term simulations (Coco et al., 2013; Roelvink, 2006). The hydrodynamic time step is set to 0.5 min so that the morphological timestep is 15 min. The total simulation period is 250 morphodynamic years including an initial period of 150 years without SLR, followed by 100 years with SLR. All combinations of the key environmental conditions, namely SLR rates, tidal ranges, waves, sediment supply and coastal slopes were simulated to study the responses of mangrove forests (Table S3 in Supporting Information S1; more detailed model parameter settings can be found in Tables S1 and S2 of Supporting Information S1).

## 3 Results

Section 3.1 provide an overview of the coastal and mangrove development throughout the parameter space, that is, varying tides, waves and sediment supply without SLR and then Section 3.2 show results of coastal and mangrove development with various rates of SLR. Subsequently Section 3.3, we compare sediment budget changes between the different scenarios introduced in Sections 3.1 and 3.2.

### 3.1 Mangrove Behaviors Under Varying Environmental Conditions: Without SLR

**Figure 3:**
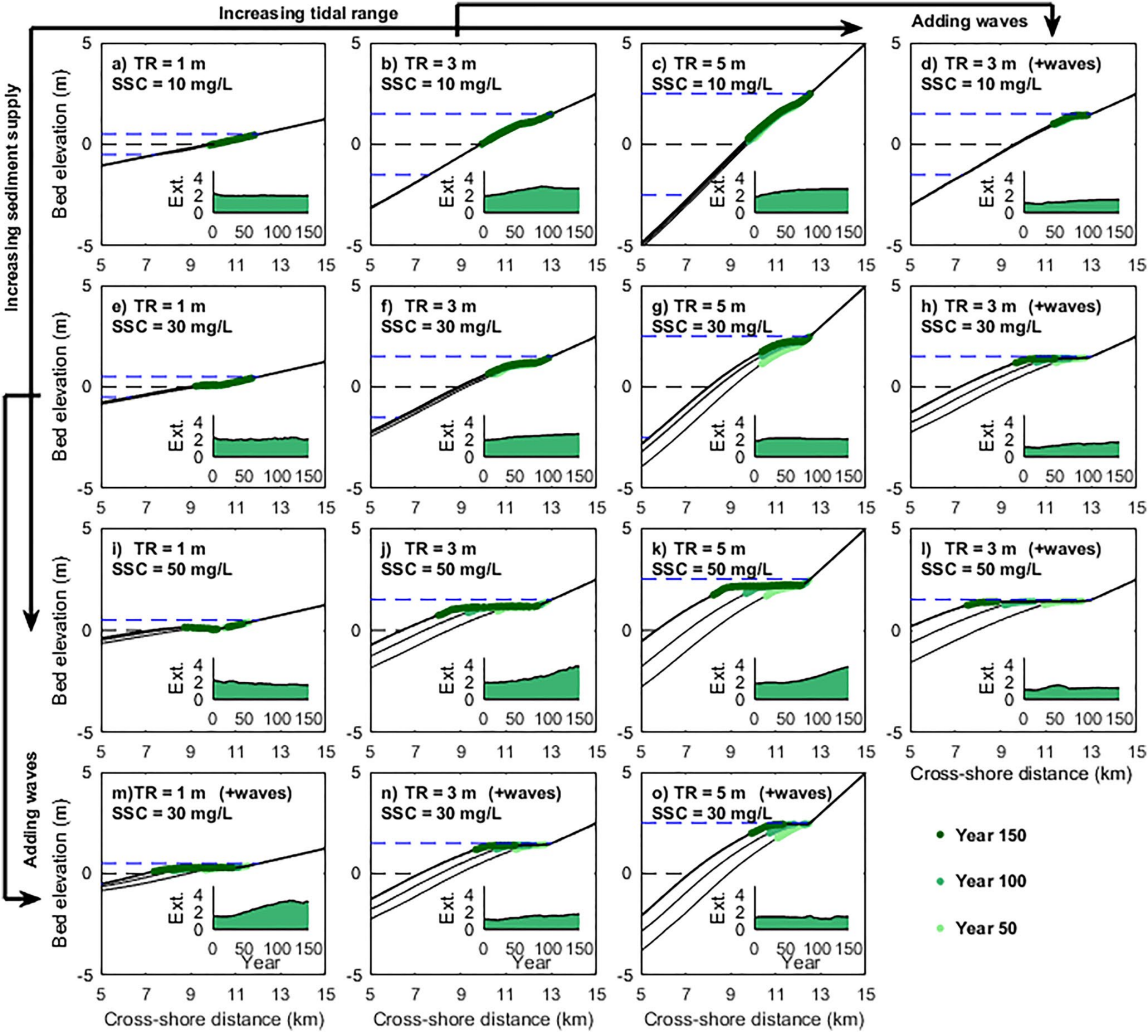
Mangrove development in response to different combinations of tidal range, sediment supply, wave action and coastal slope. Results from the first to the third column and the first to the third row indicate mangrove responses to increasing tidal range and sediment supply, respectively. Comparison results with wave effects are added with increasing tidal ranges in the fourth row (m–o) and with increasing sediment supply in the fourth column (d, h, and l). The evolution of coastal profile and mangrove forest are shown after 50, 100, and 150 years. Green colors indicate mangrove presence, with increasingly darker shades representing temporal evolution. The black dashed line and its adjacent two blue dashed lines above and below represent mean water level, high water level and low water level, respectively. The inserts show temporal changes in the horizontal extent of mangrove forests (abbreviated as Ext., unit: km). TR and SSC represent the tidal range and external suspended sediment concentration applied at the seaward boundary, respectively.

Accretion along the profile can be enhanced by increasing tidal range, sediment supply or adding wave effects, while changes in the mangrove seaward edge are modulated by the balance of tidal range and sediment supply, and waves will further limit mangrove seaward colonization around the mean water level (Figure 3).

**Figure 4:**
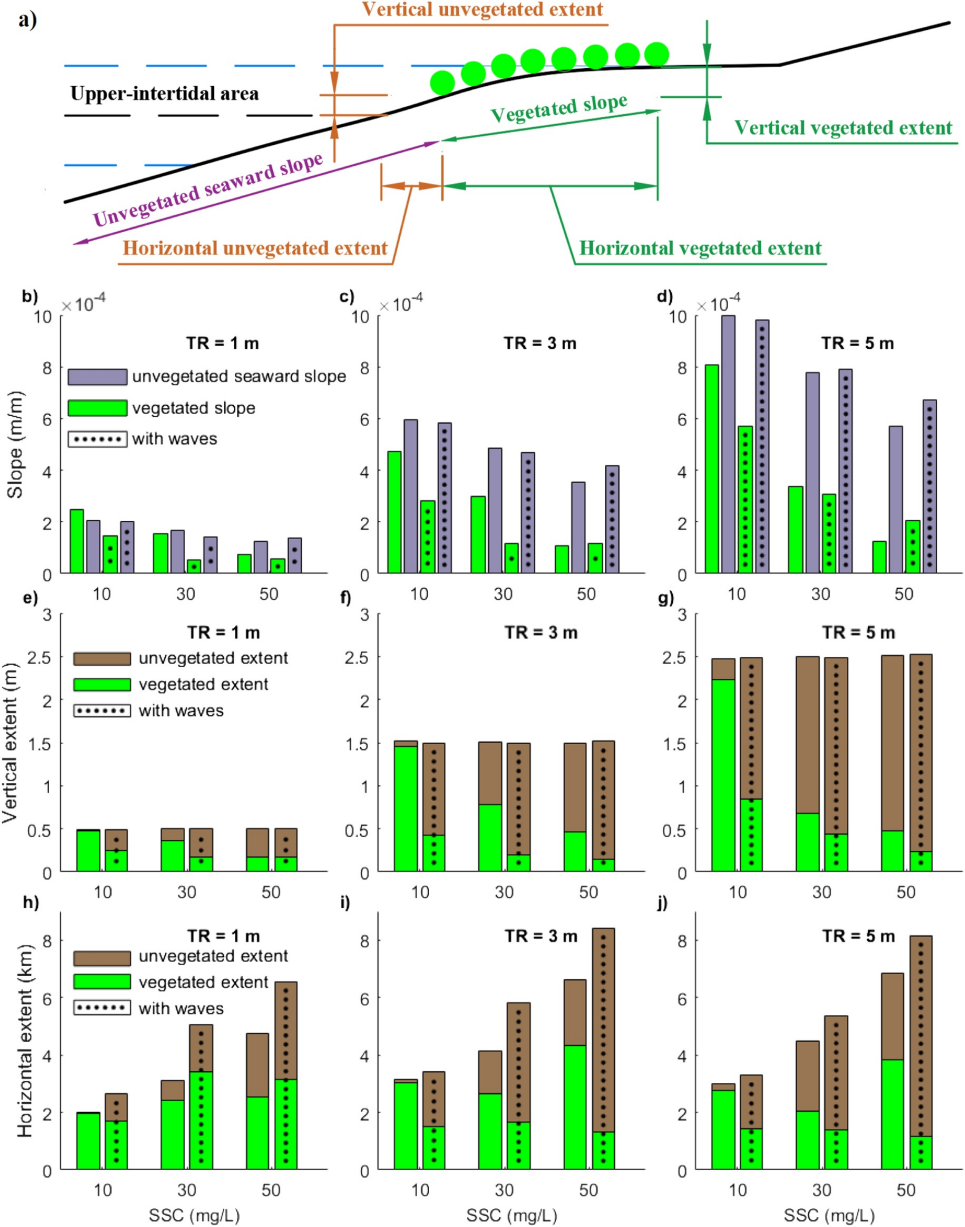
Comparison of slope and extent between unvegetated and vegetated areas. (a) Schematic figure indicating parameters for comparison in the following subplots. (b–d) The comparison of coastal slope between seaward unvegetated area (purple bars, measured from seaward boundary to mangrove seaward edge) and vegetated area (green bars, measured from mangrove seaward edge to landward edge). (e–g) The comparison of the vertical extent of unvegetated area within the upper‐intertidal area (brown bars, measured from mean water level to mangrove seaward edge) and vegetated area (green bars, measured from mangrove seaward edge to landward edge). Thus, the vertical vegetation extent refers to the elevation range over which mangroves exist. (h–j) The comparison of the horizontal extent of unvegetated area within the upper‐intertidal area (brown bars, measured from mean water level to mangrove seaward edge) and vegetated area (green bars, measured from mangrove seaward edge to landward edge).

Low sediment supply (10 mg/L) resulted in small changes in the cross‐shore profile among different tidal systems (micro‐, meso‐, and macro‐tidal systems), where vegetation remained relatively stable covering nearly the complete upper‐intertidal area, with vegetation occupying nearly 100%, 100%, and 90% of upper‐intertidal flats in micro‐, meso‐, and macro‐tidal systems, respectively (Figures 3a–3c). Vegetation extent was mainly governed by the vertical and horizontal extent of the upper‐intertidal area, which was determined by the tidal range and its corresponding coastal slope (Figures 4b–4j; 10 mg/L without waves). Vertical vegetation extent under larger tidal range conditions (∼1.5 and 2.2 m in meso‐ and macro‐tidal systems, respectively) was nearly 3 or 4 times the extent in micro‐tidal systems (∼0.5 m; Figures 4e–4g; 10 mg/L without waves), but their horizontal extent (∼3.0 and ∼2.8 km in meso‐ and macro‐tidal systems, respectively) was only slightly larger than in micro‐tidal systems (∼2 km; Figures 4h–4j; 10 mg/L without waves).

Accretion along the profile was enhanced under intermediate sediment supply (30 mg/L), leading to both coastal propagation and the gradual development of a platform (Figures 3e–3g). The size of the platform increased with increasing tidal range or sediment supply. Larger sediment supply elevated the platform closer to high water level compared to lower sediment supply conditions (e.g., Figures 3c, 3g, and 3k). Thus, vegetated slopes with either larger tidal ranges or sediment supply developed gentler slopes significantly differing from the unvegetated seaward slope (Figures 4b–4d; 30 or 50 mg/L without waves). Seaward mangrove colonization with larger tidal ranges is constrained to higher elevations above mean water level (Figures 3f and 3g), resulting in a comparable (∼2.6 km in meso‐tidal systems) or smaller horizontal vegetation extent (∼2.0 km in macro‐tidal systems) than in micro‐tidal range system (∼2.4 km) under intermediate sediment supply (Figures 4h–4j; 30 mg/L without waves). Under high sediment supply, the formation of the platform elongated cross‐shore mangrove presence, resulting in a larger horizontal vegetation extent (Figures 3j and 3k), especially for systems with a larger tidal range (∼4.3 and ∼3.9 km in meso‐ and macro‐tidal systems, respectively) compared with systems with a micro‐tidal range (∼2.6 km; Figures 4h–4j; 50 mg/L without waves). Accompanied with increasing platform development for meso‐ and macro‐tidal systems, the vertical proportion of the upper‐intertidal area covered by mangroves drastically decreased, especially under high sediment supply (Figures 3j and 3k), making the vertical extent between the different tidal systems nearly equal (0.2–0.5 m; Figures 4e–4g; e.g., 50 mg/L without waves).

The presence of waves contributed to coastal platform development under intermediate and high sediment supply (30 and 50 mg/L; Figures 3h and 3l), elevating the coastal platform closer to high water level (Figures 3m and 3n). Accretion along the profile was limited with less platform development under low sediment supply (10 mg/L) so vegetation remained relatively stable (Figure 3d). Apart from the high sediment supply simulations, the contribution of waves on platform formation and coastal propagation made the vegetated slope gentler, causing a larger slope difference compared with the offshore unvegetated area (Figures 4b–4d; with waves). Vertical and horizontal vegetation extent generally decreased when waves are included, especially in systems with larger tidal ranges and sediment supply (Figures 4e–4g and 4h–4j; with waves). This caused the vertical vegetation extent to remain nearly similar (0.2 ± 0.05 m) at high sediment supply (Figures 4e–4g; 50 mg/L with waves), but horizontal vegetation extent in micro‐tidal systems (∼3 km) was nearly double or triple than in the larger tidal systems (∼1.3 and 1 km in meso‐ and macro‐tidal systems, respectively; Figures 4h–4j; 50 mg/L with waves). Since the presence of waves exerted a more evident role in the reduction of the horizontal vegetation extent in the larger tidal range systems and profile evolution was small under low sediment supply, mangrove horizontal extent remained similar (1.4–1.7 km) among different tidal range systems (Figures 4h–4j; 10 mg/L with waves).

### 3.2 Mangrove Behaviors Under Varying Environmental Conditions: With SLR

**Figure 5:**
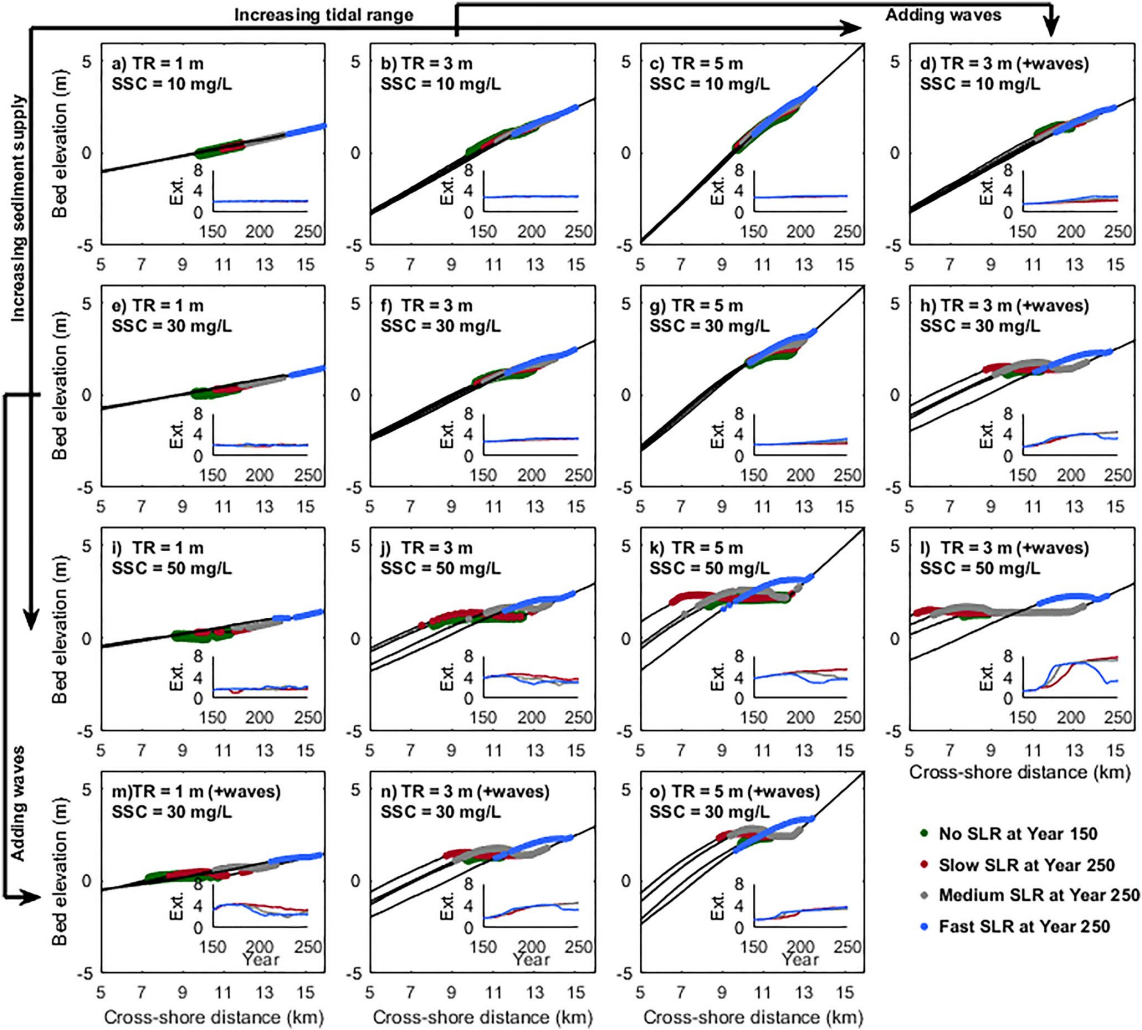
Mangrove behaviors in response to different rates of sea‐level rise (SLR). Results from the first to the third column and the first to the third row indicate mangrove responses to increasing tidal range and sediment supply, respectively. Comparison results with wave effects are added with increasing tidal range in the fourth row (m–o) and with increasing sediment supply in the fourth column (d, h, and l). Green dots show vegetation distribution before SLR, which corresponds to the results from the vegetated profile in year 150 without SLR effects (Figure 3). Red, gray and blue dots indicate mangrove forests after 100‐year slow, medium and fast SLR effects, respectively. The inserts of each Figure represent the temporal changes of mangrove horizontal extent over 100 years under different SLR effects (abbreviated as Ext., unit: km). A more detailed overview of mangrove behaviors can be seen in Figure S4 of Supporting Information S1.

Our results illustrated mangroves in larger tidal range systems with higher sediment supply were able to survive or even expand seaward with slow or medium rates of SLR (Figure 5). Mangrove development in micro‐tidal systems was greatly dependent on the rates of rising sea levels, which caused a persistent retreat of mangrove forests on a relatively stable profile with only minor changes in vegetation extent due to increased sediment supply (Figures 5a, 5e, and 5i). In contrast, mangrove behaviors in larger tidal systems were more complex. Under low sediment supply (10 mg/L), the coastal profile experienced little accretion and mangroves kept a constant extent by landward transgression as sea level was rising (Figures 5b and 5c). In larger tidal systems (meso—macro tidal), although accretion across the profile was little under intermediate sediment supply (30 mg/L), the vegetation at the seaward edge remained stable with little landward transgression under slow/medium SLR rates (Figures 5f and 5g). Interestingly, during the fast SLR, the landward retreat of the seaward vegetation edge could be observed in meso‐tidal systems (Figure 5f), however, the seaward edge remained stable in macro‐tidal systems (Figure 5g). Under high sediment supply (50 mg/L), mangroves were able to propagate seaward under slow SLR rates, whereas high SLR rates led to a landward shift of the coastal profile (i.e., seaward erosion and landward accretion along the profile) and mangrove landward transgression (Figures 5j and 5k).

The effects of waves on mangrove responses were enhanced in larger tidal range or sediment supply conditions and adjusted by the rates of SLR (Figure 5). Under low sediment supply (10 mg/L), limited accretion along the profile occurred and waves had little effect so that the mangrove seaward edge retreated as sea level increased (Figure 5d). Under intermediate to high sediment supply (30–50 mg/L), waves contributed to sediment accretion under slow/medium SLR, enhancing coastal progradation and mangrove seaward expansion (Figures 5h and 5l). However, mangrove seaward edge retreated substantially when the SLR was fast. Meanwhile, among different tidal systems, waves helped to maintain a coastal platform under slow/medium SLR, and thus the vegetation extent increased, especially in larger tidal range systems (Figures 5m–5o). Similar to the behaviors observed in the scenarios without wave effects (Figures 5e–5g; 30 mg/L), fast SLR caused mangrove seaward edge to retreat landward in micro/meso‐tidal systems but in macro‐tidal systems this seaward edge remained stable (Figures 5m–5o; 30 mg/L).

**Figure 6:**
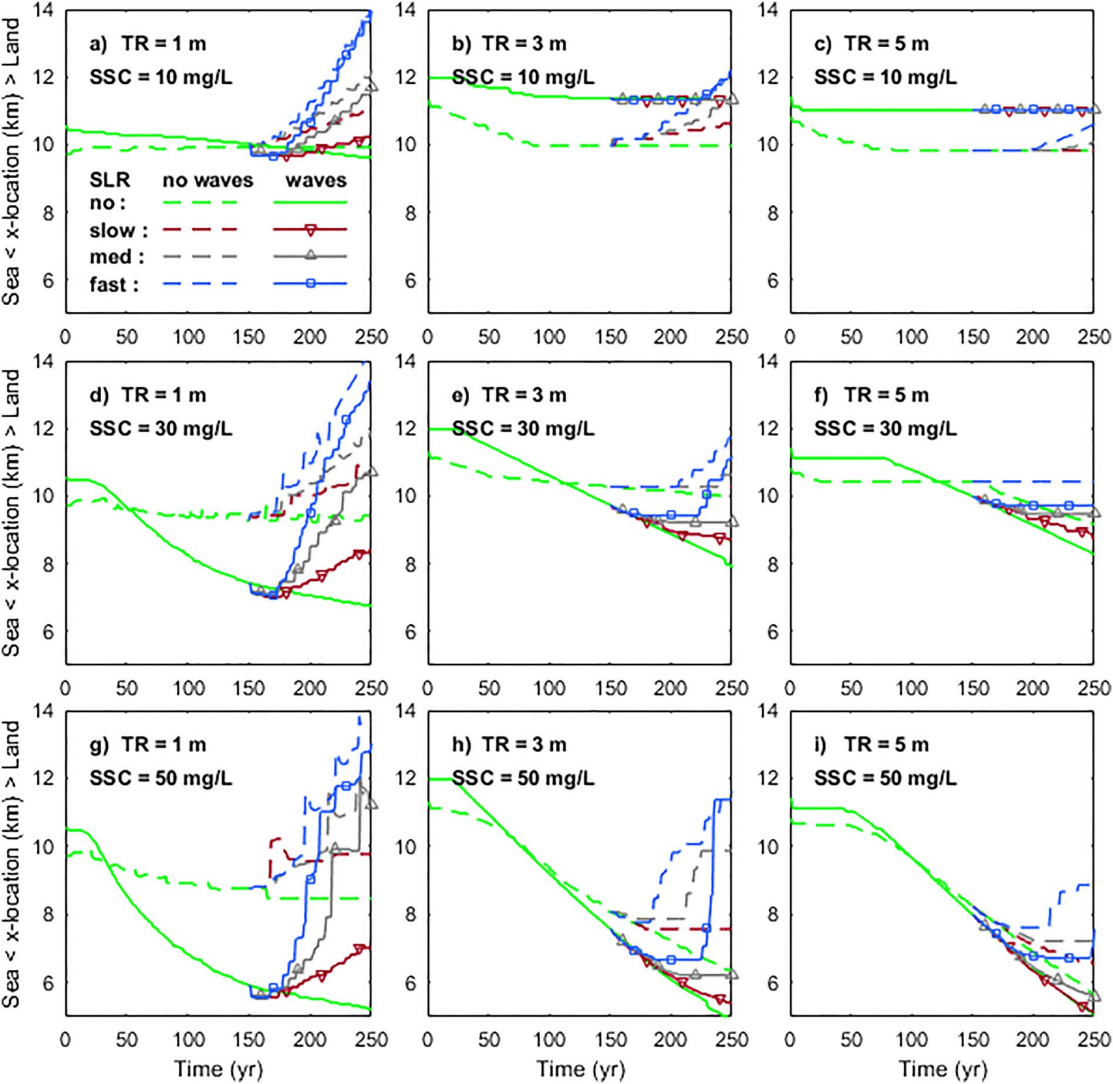
The movement of mangrove seaward edge without sea‐level rise (SLR) effects (green lines) and with three different SLR scenarios. *Y*‐axis includes two opposite directions, land and sea, showing the direction and distance over which the seaward mangrove edge moves. Increasing values represent landward retreat of the mangrove seaward boundary, while decreasing values represent seaward mangrove expansion. Red, gray, and blue lines are used to represent the results of slow, medium (abbreviated as med in a) and fast SLR. SLR was implemented after 150 years. Dashed and solid lines are used to indicate the simulations without waves and with waves, respectively.

To identify the vulnerability of mangroves during various rates of SLR, we quantified and compared the movement of the seaward mangrove edge among different environmental conditions. Our results showed that mangroves in micro‐tidal range systems were more sensitive to SLR and even slow SLR caused a substantial landward retreat, while in larger tidal range systems mangroves may even expand seaward despite a rising sea level (Figure 6). Also, mangrove seaward edge in the larger tidal range was more dependent on the balance between sediment supply and SLR rates. Low sediment supply and fast SLR rates would cause mangroves to retreat landward (blue‐dashed lines in Figures 6b and 6c), while high sediment supply and slow SLR resulted in mangrove seaward expansion (red‐dashed lines in Figures 6h and 6i). The role of waves on mangrove seaward movement differed with tidal range and SLR scenarios. In the absence of SLR effects, waves enhanced mangrove seaward expansion, especially when the tidal range was small (Figure 6). However, when sea level started to rise, waves caused mangroves to retreat faster in micro‐tidal systems (solid lines with symbols in Figures 6a, 6d, and 6g). In larger tidal range systems, the presence of waves could initially stabilize mangrove seaward edge even under fast SLR. Still, mangrove retreat may then be again faster when sea level was rising (e.g., blue‐solid line in Figure 6h).

### 3.3 Sediment Budget Changes Under SLR

**Figure 7:**
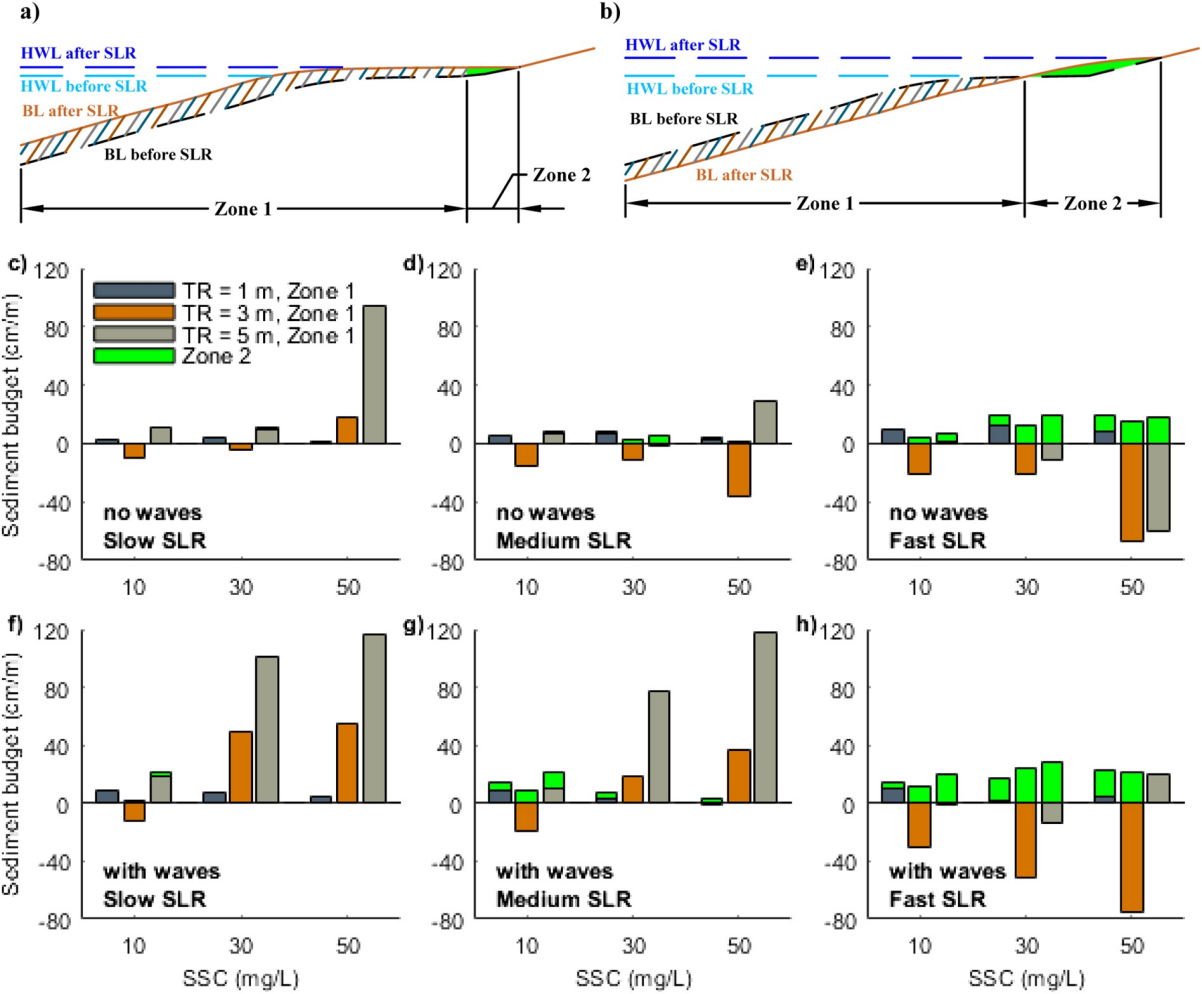
Sediment budget changes (converted to cm/m, representing vertical changes in profile elevation per meter in the cross‐shore direction) during sea‐level rise (SLR) under various coastal conditions. The sediment budget is calculated here as the sum of bed level differences in two respective zones (Zone 1 and Zone 2) within the 100‐year SLR period and divided by their corresponding horizontal lengths. (a and b) Schematic figures indicating Zone 1 and Zone 2 in two different scenarios: (a) coastal propagation where there is deposition in Zone 1 and limited deposition in Zone 2 and (b) coastal retreat where there is erosion in Zone 1 and substantial deposition in Zone 2. HWL and BL here represent high water level and bed level, respectively. Panels (c–e and f–h) represent the results without wave effects and with wave effects, respectively. Panels (c and f), (d and g), and (e and h) are the results under slow, medium, and fast SLR, respectively. Blue, brown and gray bars are used to indicate sediment budget changes in Zone 1 and green bars are used to indicate changes in Zone 2.

Finally, we analyzed how SLR altered sediment budgets and how this is dependent on coastal conditions. Since the amount of sediment accumulated and displaced along the profile and in intertidal zones during rising sea levels is important for mangroves to maintain their relative elevation in the tidal frame, we evaluated sediment budget changes over two distinct sections: (a) over the active profile up to the high‐water level before SLR (HWL before SLR; Zone 1 in Figures 7a and 7b) and (b) over the profile section that became flooded during SLR (between HWL after and HWL before SLR; Zone 2 in Figures 7a and 7b) as the latter represented the potential new accommodation space available for mangroves. Under slow SLR rates and in the absence of waves, the sediment is mainly deposited in Zone 1 (Figure 7c). The amount of sediment accumulated in this zone in micro‐tidal systems remained similar (∼3 cm/m) despite changes in sediment supply (blue bars in Figure 7c), while in larger tidal range systems sediment accumulation in Zone 1 increased as sediment supply increased (brown or gray bars in Figure 7c). Under medium SLR rates, there was a slight increase in sediment deposition in Zone 1 for micro‐tidal systems (blue bars in Figure 7d), while more sediment erosion occurred in meso‐tidal systems (brown bars in Figure 7d) and sediment deposition became less in macro‐tidal systems (gray bars in Figure 7d). Meanwhile, some sediment started to be deposited in Zone 2 in the systems with either a larger tidal range or sediment supply (green bars in Figure 7d). Under fast SLR rates, more sediment was deposited in Zone 1 in micro‐tidal systems (blue bars in Figure 7e), while in larger tidal range systems this profile section (Zone 1) was further eroded, especially when sediment supply was large (brown or gray bars in Figure 7e). Sediment accumulation in Zone 2 increased for all tidal range systems under such high SLR rates (green bars in Figure 7e). The presence of waves generally enhanced sediment deposition in Zone 1 when the SLR was slow/medium (Figures 7f and 7g). Also, more sediment was deposited in Zone 2 when sediment supply was low (green bars in Figures 7f and 7g). Under fast SLR, with the exception of macro‐tidal systems with high sediment supply, waves contributed to enhancing sediment deposition in Zone 2 (green bars in Figure 7h), but they also reduced sediment deposition or caused more sediment erosion in Zone 1 (e.g., blue or brown bars in Figure 7h).

## 4 Discussion

Our numerical scenarios highlight that coastal conditions non‐linearly influence mangrove behavior and determine their vulnerability to SLR. The analysis covers variations in tides, wave action, sediment supply and coastal slope, under different SLR scenarios, indicating distinct mangrove responses to SLR across various coastal systems. In this section, we discuss (Section 4.1) how different coastal conditions control profile evolution and mangrove dynamics, (Section 4.2) the key factors determining mangrove vulnerability to SLR, and (Section 4.3) other relevant processes shaping vegetated coastal profiles.

### 4.1 Key Controlling Factors Influencing the Changes in Coastal Profile Evolution and Mangrove Dynamics

#### 4.1.1 Effects of Coastal Conditions on Shaping Vegetated Coastal Profiles

This study aims to unravel the controlling processes driving changes in mangrove forests and morphological evolution through idealized numerical simulations and, as such, modeling behaviors and trends become the main concern (Murray, 2013). Consistent with natural mangrove systems (Figure 1), the model predicts variations in profile shapes and vegetation distributions in response to different coastal environmental conditions. In our model as well as in field observations, linear intertidal profile shapes occur in systems with either small tidal range or low sediment supply, while increasing these two variables leads to the formation of a gently sloping vegetated platform occupied by mangroves (Figures 1, 3, and 4b–4d) (Bryan et al., 2017; Nardin et al., 2021; Semeniuk, 2018). The elevation of the vegetated platform tends to move closer to the high water level when sediment supply increases (e.g., Figures 3c, 3g, and 3k), which is in agreement with previous field observations and numerical experiments (Goodwin & Mudd, 2020; Kirwan et al., 2016; Swales et al., 2019). The comparison between different scenarios reveals that tidal range and sediment supply play a determining role on the overall coastal slope and mangrove extent with relatively gentle/steep profiles existing under small/large tidal range and high/low sediment supply, respectively (Figures 4b–4d). These trends are consistent with modeling efforts of unvegetated tidal flats (Friedrichs, 2011). Additional small wind waves enhance accretion and coastal progradation in all our simulations (Figure 3) as a result of the enhanced onshore sediment fluxes (Figures S5 and S6 in Supporting Information S1), which has also been observed in the field (Swales et al., 2019). The role of small wind waves on sediment transport varies with the coastal condition (i.e., tidal range and associated coastal slope; Figures S5 and S6 in Supporting Information S1). In micro‐tidal systems, the presence of waves is able to shift a stable profile (e.g., Figure 3e) to a propagating profile (e.g., Figure 3m), promoting mangroves seaward expansion (Figures 6a, 6d, and 6g) and increasing horizontal vegetation extent (Figure 4h) due to the shallower water depth and weaker currents (Carniello et al., 2011). In meso‐ and macro tidal systems, the presence of waves limits seaward vegetation colonization under low sediment supply due to the enhanced hydrodynamic forces and limited coastal propagation (Figures 3b and 3d); on the contrary, under intermediate and high sediment supply, waves further increase existing progradation (Figures 3f, 3h, 3j, and 3l). This is in agreement with previous research that small waves (less than 20 cm) are capable of resuspending sediments over tidal flats (Green, 2011; Green et al., 1997; Uncles & Stephens, 2000; van der Wegen et al., 2017) and thereby potentially nourishing vegetated habitats (Carling, 1982). Thus, our simulations are able to highlight that tidal range, waves and sediment supply impact bio‐physical feedbacks between vegetation extent (either in the horizontal or vertical dimension) and coastal slope. The complexity of these non‐linear feedbacks further governs coastal landscape configuration and vegetation distribution, leading to different degrees of platform formation which have not been identified so far (Figures 4e–4g and 4h–4j).

#### 4.1.2 Effects of Hydrodynamic Forcing and Profile Evolution on Mangrove Colonization

**Figure 8:**
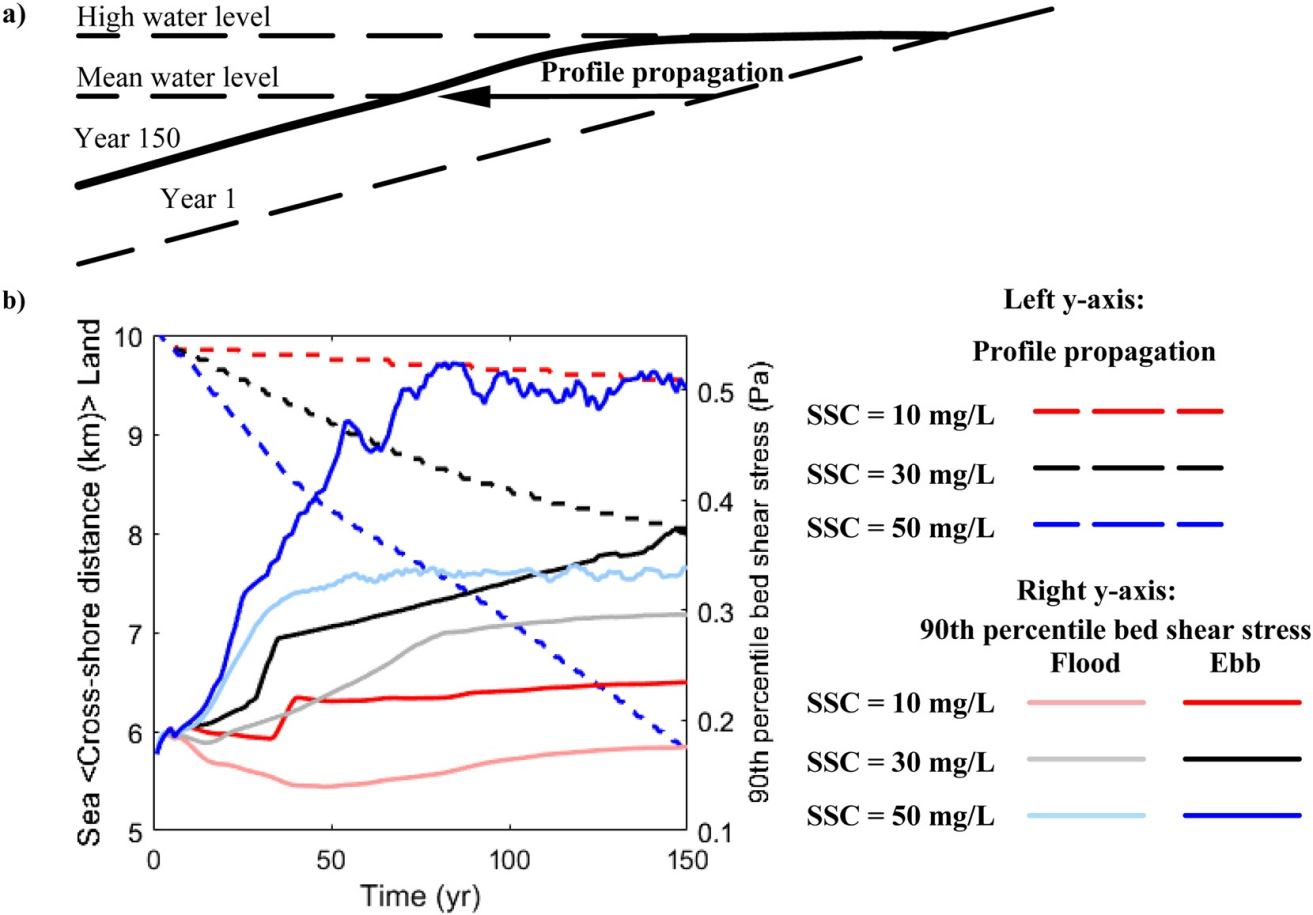
Relations between coastal profile propagation and bed shear stress changes. (a) Schematic figure illustrating profile propagation from year 1 to year 150. (b) Relationships between coastal profile propagations (left *y*‐axis) and bed shear stress (right *y*‐axis) in the macro‐tidal systems without waves before sea level rises (Figures 3c, 3g, and 3k). The changes in cross‐shore distance (dashed lines) and the corresponding flood/ebb bed shear stress (solid lines) in panel (b) are based on the intersection between coastal profile and mean water level, as indicated by the arrow in (a). Red, black, and blue colors are used to show different scenarios with sediment supply of 10, 30, and 50 mg/L, respectively. Light color shades of solid lines indicate bed shear stress during the flood period and dark color shades indicate bed shear stress during the ebb period.

Tidal range and sediment supply have been identified as key factors determining mangrove distribution (Ellison, 2015). Mangrove extent has been linearly linked with tidal range (Ellison, 2015; Lovelock et al., 2015), however, our results show that vegetation extent (either in the horizontal or vertical dimension) varies non‐linearly with changes in tidal range and sediment supply which results in unexpected responses compared to the first‐order approximation (Figures 4e–4g and 4h–4j). This is linked to the conditions inhibiting mangrove colonization. Previous studies suggested that the establishment and survival of mangrove seedlings are constrained by high bed shear stress even under an appropriate inundation regime, and thus linked to so‐called windows of opportunity (WoO) governed by variations in external forcings (Balke et al., 2011; Friess et al., 2012). Aligned with these studies, here we show, using constant hydrodynamic forcing (i.e., M2 tide, regular sediment supply and waves), that increased tidal range (i.e., stronger tidal currents) or additional wave action limits mangrove seaward colonization (e.g., Figures 3f, 3g, and 3o), as is also observed at the Firth of Thames, New Zealand (Balke et al., 2015). Comparisons on the seaward edge elevation of mangrove forests between natural systems and our model simulations across a variety of tidal ranges confirm our model finding that mangrove elevations generally increase with tidal range (Figure S7 and Table S4 in Supporting Information S1). Moreover, our modeling results indicate that altered tidal currents in response to platform morphological adaptation further limit the colonization. More specifically, in macro‐tidal systems, profile reconfiguration and platform formation enhance ebb tidal currents (Figure 8), increasing bed shear stresses and limiting vegetation colonization at the mid‐intertidal moving mangroves toward higher bed elevations (Figures 3g and 3k). Our simulations thus indicate that mangrove colonization can be linked to profile reconfiguration through bio‐morphodynamic feedbacks. As a consequence of these colonization constraints, the lower elevation limit for mangroves can be higher than mean sea level (e.g., Figures 3g and 3k), which has also been observed in the field (Balke et al., 2014; Bryan et al., 2017; Swales et al., 2021). In addition to capturing this behavior, we show that coastal environmental condition plays a key role in determining the lower limit mangroves can colonize in the intertidal.

### 4.2 Feedbacks Between Coastal Conditions, Mangrove Vulnerability, and SLR

#### 4.2.1 On the Role of Tidal Range, Waves, Sediment Supply, and Coastal Slope

Previous research illustrates that the balance between SLR rates and vertical accretion rates determines the resilience of mangroves against drowning (Woodroffe et al., 2016). As previously identified, external sediment supply plays a major role in driving vertical accretion rates (Lovelock et al., 2015). Simulations for meso‐ and macro‐tidal systems with fast SLR rate and low sediment supply caused relatively stable coastal profiles over which mangroves transgressed landward (Figures 5b and 5c). However, an increase in sediment supply and reduction in SLR rate led to profile seaward propagation and thus mangrove expansion (Figures 5j and 5k). This reinforces the importance of the balance between SLR and sediment supply, at least under these tidal conditions. Yet, our simulations also show that sediment accretion rates are not necessarily enhanced by increased sediment supply, but may instead depend non‐linearly on environmental conditions, such as tidal range, coastal slope and waves (Figure 7). Thus, changes in mangrove extent under SLR may vary for systems with varying tidal ranges despite similar sediment supplies (e.g., Figures 5i, 5j, and 5k). More specifically, mangroves in micro‐tidal systems are found to rapidly retreat landward even under slow SLR and high sediment supply (Figures 5i and 6g). The potential reasons are (a) the gentler coastal equilibrium slope in micro‐tidal system (Figure 4b), where a small water level change will cause a large horizontal displacement of the inundation regime and thus strongly affect mangrove survival and (b) the lower ability to capture sediment in micro‐tidal systems where sediment deposition over the profile remains limited so that vertical accretion cannot allow mangroves to maintain their relative elevation within the tidal frame (Figure 7). In addition, since the presence of small wind waves enhances accretion and seaward profile propagation, mangrove seaward expansion is still possible under slow/medium SLR, compared to the results without wave effects (Figures 5h, 5l, 5n, and 5o). This implies that when small waves are present, the extra sediment stirring and resultant transport fluxes can play a role in enhancing the resilience of mangrove systems under SLR, which confirms earlier research showing the effects of waves on critical SLR rates in salt marsh systems (FitzGerald & Hughes, 2019; Schuerch et al., 2013).

#### 4.2.2 Effects of Inundation Buffer

**Figure 9:**
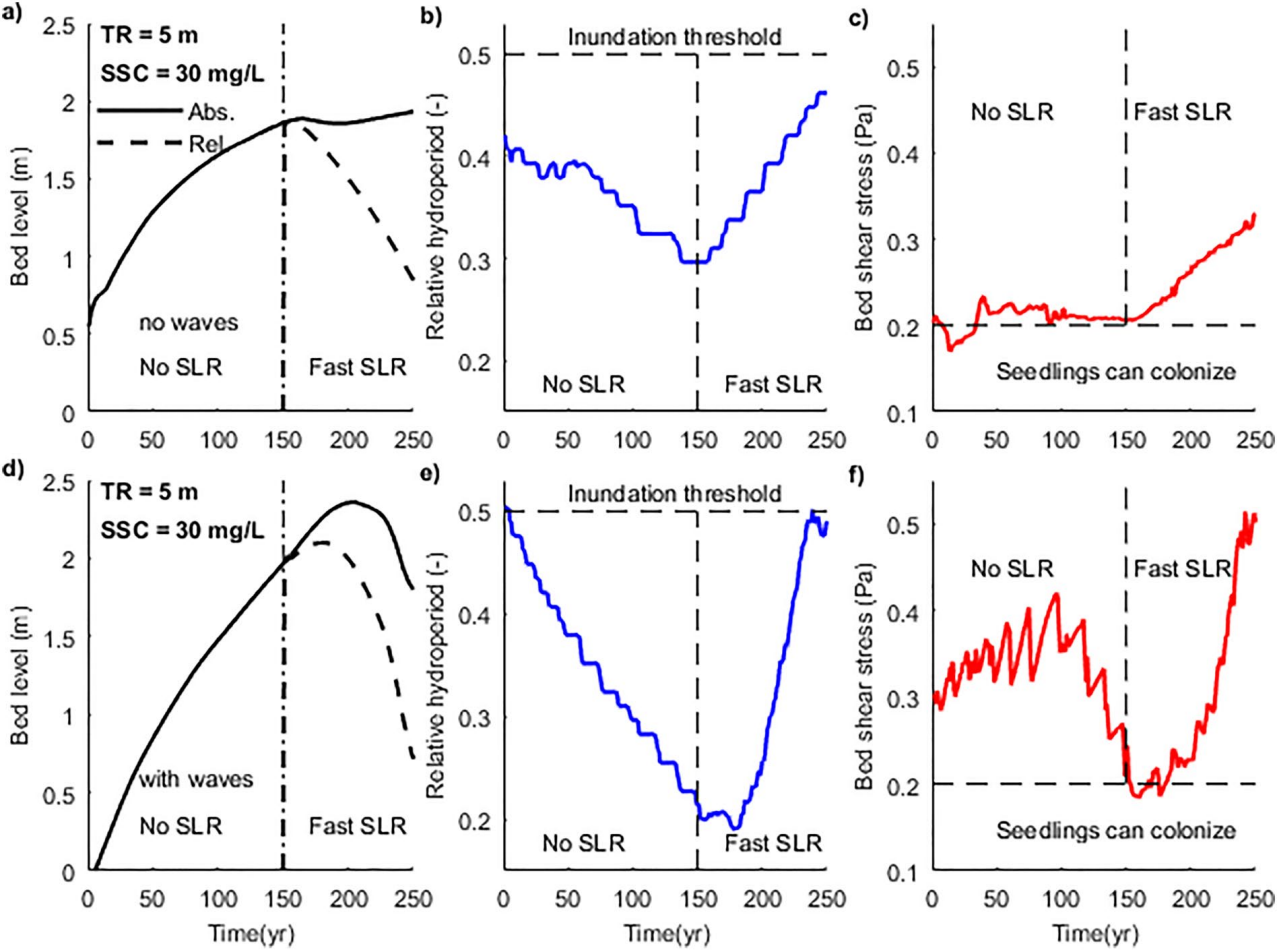
Temporal changes of key parameters over 250 years. The scenarios represent a stable seaward vegetation edge during fast sea‐level rise (SLR) under intermediate sediment supply (30 mg/L) and large tidal range (5 m) (Figures 5g and 5o). SLR starts at year 150. The location for analysis is the seaward edge of mangrove forests at the beginning of SLR. These mangroves can subsequently survive fast SLR because of an inundation buffer. Panels (a–c) are results without waves and (d–f) with waves. Temporal changes of (a and d) absolute bed level (solid lines) and relative bed level (to mean water level; dashed lines), (b and e) relative hydroperiod, and (c and f) 90th percentile of bed shear stress.

Our modeling results suggest that the mangrove seaward edge can remain stable even under fast SLR and no or limited sediment accretion (Figures 5g and 5o). This is because colonization restrictions can cause mangroves to establish at high elevations with an initially low hydroperiod, and thus potentially withstand increasing tidal inundation. This effectively creates an inundation buffer before SLR, such that the inundation threshold of mangrove trees is not immediately exceeded during rising sea levels (Figure 9). This can be exemplified by focusing on the changes in environmental parameters at the seaward edge of mangrove forests when SLR starts. Before SLR, the bed level is increasing which corresponds to a decreasing relative hydroperiod (Figures 9a, 9b, 9d, and 9e). When waves are absent, mangroves are able to colonize as soon as both hydrodynamic forces and relative hydroperiod are below the thresholds (at ∼year 10; Figures 9b and 9c). Excess bed shear stress induced by tides limits further seaward mangrove expansion despite ongoing coastal progradation (Figure 3g). Although the mangrove seaward edge thus remains stable, the inundation regime becomes more favorable (relative hydroperiod decreases from 0.4 to 0.3; Figure 9b). When waves are present, the excess bed shear stress is more profound and hinders mangrove colonization even though the inundation regime is highly suitable (Figures 9e and 9f). As such, when colonization eventually takes place (at ∼year 150), mangrove presence is confined to higher elevations, where the relative hydroperiod is well below the inundation threshold (relative hydroperiod is approximately 0.2; Figure 9e). Thus, the seaward mangrove forest edge prior to SLR (either with waves or without waves) is located at an elevation and inundation regime which constitutes an inundation buffer.

When SLR occurs, increasing bed shear stress limits accretion along the profile prohibiting bed levels to keep up with SLR, resulting in an increase in relative hydroperiod (Figures 9b and 9e). However, the inundation regime at the seaward mangrove edge still remains below the vegetation mortality threshold (Figures 9b and 9e) so that mangroves are able to persist under such fast SLR rates. This implies that the capacity of mangroves to survive SLR is not only based on the balance between SLR rates and vertical accretion rates which has been widely recognized (Woodroffe et al., 2016), but also based on the relative bed elevation occupied by mangroves, which in turn is controlled by vegetation colonization and morphology feedbacks. Although our model simplifies colonization thresholds, it clearly demonstrates that mangrove survival under SLR needs to account for complex growth dynamics. The set‐up of our simulations is based on 150 years without SLR impacts followed by 100 years with SLR. This may overestimate the established inundation buffer in comparison to natural systems evolving under continuous SLR (Lovelock et al., 2015; Saintilan et al., 2020). However, our results nevertheless demonstrate that the acceleration of SLR predicted in the near future might severely reduce the resilience of mangrove systems, rendering them unable to maintain an inundation buffer. The current study only uses a simplified M2 tide to drive tidal variations, however, in reality, differences in spring‐neap cycles, barometric pressures and storm waves will potentially increase non‐linearities of hydrodynamic conditions and thus complicate mangrove establishment (Balke et al., 2011). Including these factors goes beyond the scope of the current study, but is an important avenue for future research.

#### 4.2.3 Effects of Profile Reconfiguration and Infilling of Accommodation Space

Consistent with previous findings, the creation of landward habitats can compensate for the loss of seaward mangrove forests under rising sea levels, thus the total mangrove extent may remain stable or even increase (Figure 5) (Schuerch et al., 2018). Our model shows that mangrove landward expansion is possible without infilling of the newly generated accommodation space (e.g., Figures 5a–5c). This is because mangroves can survive within a range of relative hydroperiods (Chapman, 1976) and the coastal conditions, determined by tidal range and coastal slope, create a gradient of inundation pattern across the profile. This finding seemingly contradicts other studies which note that increasing sediment mass is needed to completely infill newly created accommodation space in order to maintain wetland area (Törnqvist et al., 2019, 2021). Yet, our centennial‐scale simulations reveal that whether limited, partial or complete infilling occurs depends on coastal conditions and the bio‐physical adaption of the coastal profile under SLR. For instance, if accretion along the profile is limited as a result of either low sediment supply or small tidal range, SLR may only shift the inundation zones landward with mangrove colonization occurring in newly created landward habitats (Figure 5). Alternatively, sediment infilling of new accommodation space may happen and in fact originate from erosion and reconfiguration of the lower coastal profile (Figure 7). This erosion is caused by the strengthening of tidal currents linked to increases in the tidal prism because of landward flooding (Figure S8 in Supporting Information S1). Meanwhile, the strengthening of tidal currents not only causes adjustments in the coastal profile, but also makes the mangroves in the seaward area particularly vulnerable (e.g., Figures 5j and 5k). This implies that profile reconfigurations, changes in mangrove coverage and infilling of accommodation space emerge from complex bio‐morphodynamic interactions. Thus, infilling of the accommodation space may not be necessary for mangrove survival, however, it can be important for long‐term sustainability and is highly dependent on bio‐physical feedbacks (Rogers et al., 2019; Törnqvist et al., 2021).

These bio‐physical feedbacks in turn strongly depend on the rates of SLR as also suggested by Fagherazzi et al. (2004). Offshore sediment erosion, onshore sedimentation and filling up of newly available accommodation space are enhanced under increasing rates of SLR (Figure 7). This is in agreement with field observations from salt marshes, showing moderate SLR causing coastal progradation, while increased SLR rates induced offshore sediment erosion and increased transport on the vegetated platform (Yang et al., 2020). Interestingly, apart from the influence of SLR rates, we find that offshore erosion (i.e., the decrease of bed level in seaward areas) and the landward shift of the coastal profile are most significant for the systems with high sediment supply (50 mg/L) in which a profound platform was formed (Figures 5k and 5l). This highlights that the established profile configuration, including the cross‐shore extent of the platform, has a major impact on mangrove responses to SLR and filling up of the accommodation space.

#### 4.2.4 Non‐Linear Relations Between SLR and Sediment Accretion Rates

**Figure 10:**
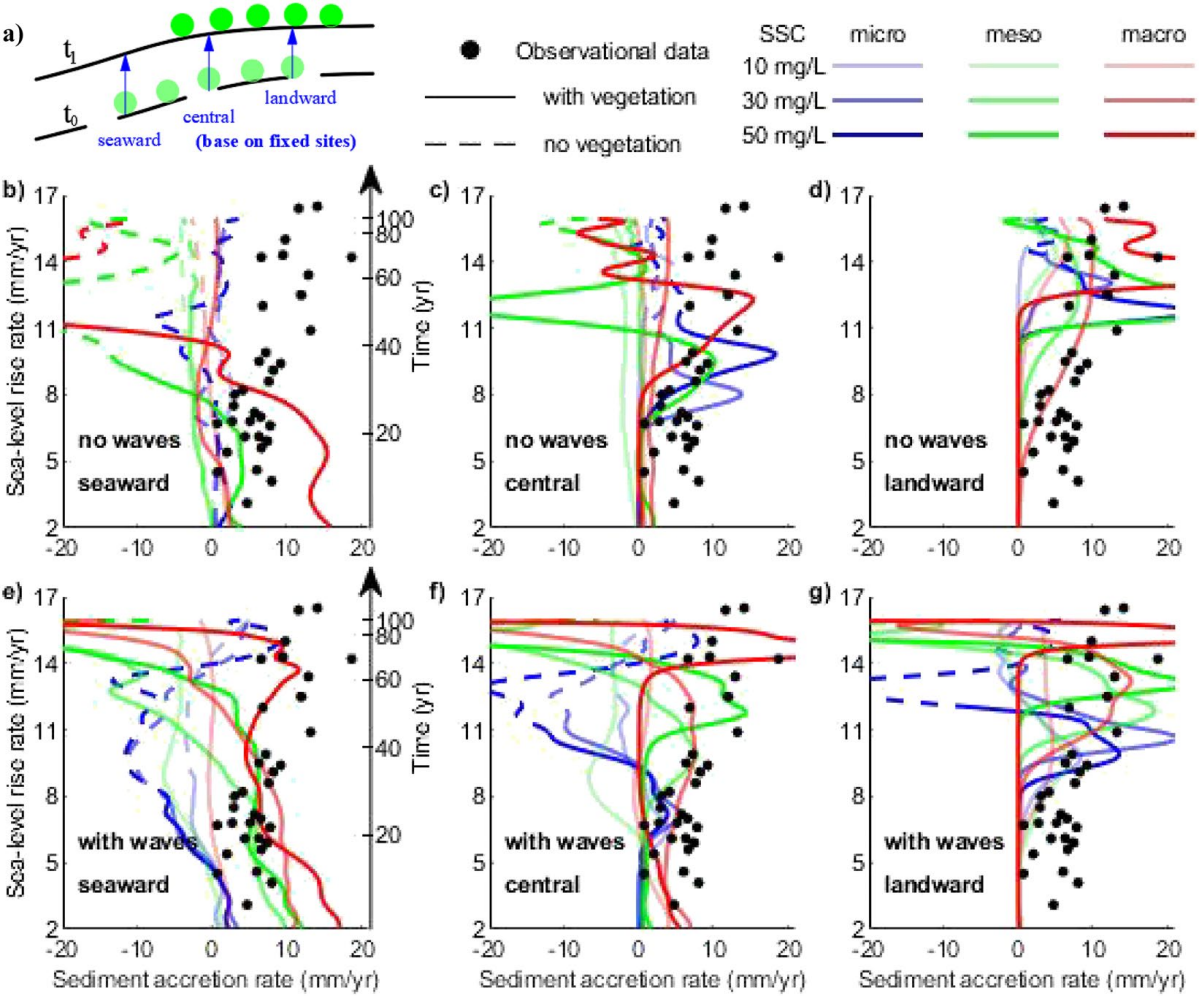
Comparison of sea‐level rise (SLR) rates and sediment accretion rates between numerical simulations (lines) and field observations (black dots, summarized by McKee et al. [2021]), evaluated for the fast SLR scenarios. Schematic figure (a) illustrates how the relation is evaluated: in the (b and e) seaward, (c and f) central, and (d and g) landward region of the initial mangrove forest. Locations used to calculate seaward, central and landward accretion rates are based on fixed sites before the SLR. Blue, green and red color lines in subplots (b–g) are used to represent the micro‐, meso‐, and macro‐tidal systems, respectively. Light, medium and dark color shades are used to represent the low (10 mg/L), intermediate (30 mg/L) and high (50 mg/L) sediment supply, respectively. The switch from solid lines to dashed lines in (b–g) represents the disappearance of vegetation at those specific locations due to SLR.

Previous research has investigated mangrove vulnerability by evaluating the relation between local SLR rate and surface accretion, as summarized by McKee et al. (2021) (black dots in Figure 10; Table S4 in Supporting Information S1). Consistent with these field observations, our model results not only capture the natural sediment accretion rates (Figure S9 in Supporting Information S1), but also show that sediment accretion rates may increase in response to increasing SLR rates (Figures 10b–10d and 10e–10g). This is because SLR enhances sediment deposition by increasing the relative hydroperiod and suspended sediment concentration over the vegetated flats under fast‐rising sea levels, in particular in larger tidal range systems (Figure S10 in Supporting Information S1). This is consistent with the current view that mangroves can keep up with SLR through bio‐physical feedbacks involving enhanced deposition under prolonged inundation (Rodriguez et al., 2017; Schuerch et al., 2018), but also shows that SLR may increase sedimentation on vegetated flats by modifying sediment availability.

The timing and magnitude of accelerations in accretion depend non‐linearly on the distance from the mangrove seaward edge (Figure 10). Strong spatial variations in accretion may initially occur across the forest, which has also been observed in the field (Swales & Lovelock, 2020; Swales et al., 2021). Our model results further suggest that such spatial variations in accretion rates are influenced by rising sea levels and vary with coastal conditions. In the absence of waves, accretion is initially low apart from in the most seaward region of the forest in the meso‐ and macro‐tidal system (dark green and red in Figure 10b). However, when the SLR rate increases, accretion starts to accelerate in the central region (Figure 10c) and then also in the more landward region, especially when sediment supply is high (Figure 10d). When waves are present, the initial accretion in the seaward region is enhanced across the majority of tidal range and sediment supply simulations (Figure 10e). Interestingly, in the central region of the forest, waves can either enhance (e.g., compare dark green lines in Figures 10c and 10f) or reduce (e.g., compare dark blue lines in Figures 10c and 10f) the acceleration in sediment accumulation. Also, SLR rates driving increasing accretion rates in the landward region are lower with waves (Figure 10d vs. Figure 10g).

The increase in accretion in the central and landward forests may coincide with reduced accretion and eventual erosion in the seaward region. This reflects the aforementioned profile reconfiguration which also results in mangrove mortality (see transitions from red and green solid lines to dashed lines in Figure 10b and from blue solid lines to dashed lines in Figure 10e). Recent paleorecord findings on mangrove vertical accretion preserved in the sedimentary archives show mangroves are very likely to be unable to initiate sustained accretion and be submerged when SLR rates exceed 6.1 mm/yr (Saintilan et al., 2020). Although the majority of our simulated scenarios show seaward mangrove mortality occurring for SLR rates below 10 mm/yr (see transitions to dashed lines in Figures 10b and 10e), our study highlights the spatial variation in the response of sediment accretion to SLR. This suggests the interpretation of paleoenvironmental records should encompass records derived along coastal gradients as critical SLR thresholds are inherently linked to the location within mangrove forest.

#### 4.2.5 Synthesizing Mangrove Vulnerability and Evaluating the Effects of Dynamic Coastal Profiles and Complex Vegetation Behavior

**Figure 11:**
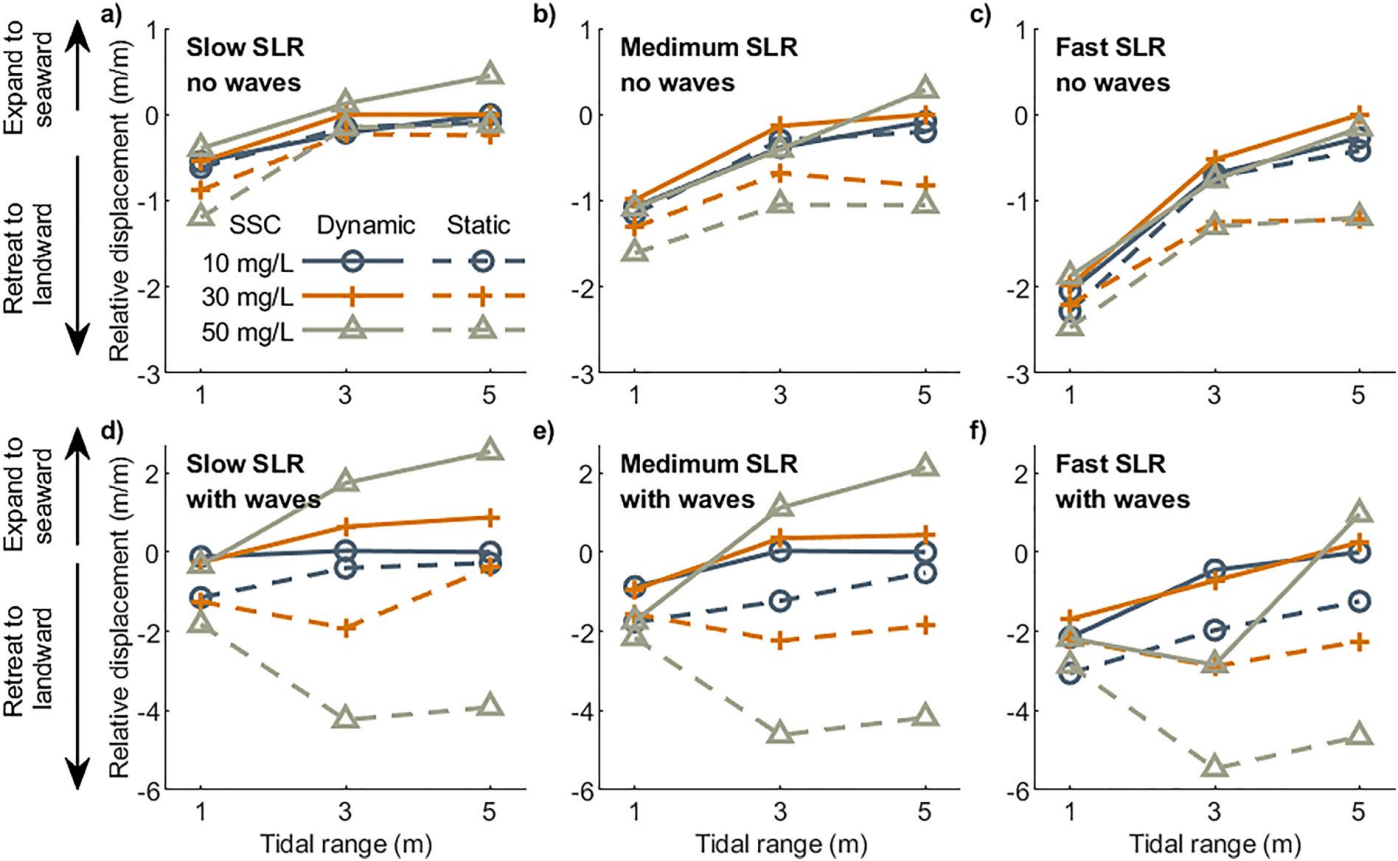
Relative displacement after 100‐year sea‐level rise (SLR) impacts. Here, relative displacement is defined as the retreat extent of mangrove seaward edge after SLR effects over the total horizontal vegetation extent before SLR. As an example, if the new mangrove seaward edge after 100‐year SLR effects is exactly the landward edge before SLR, then this “relative displacement” gets a value of −1. If it remains stable without retreat, it gets a value of 0. The top row and bottom row represent the relative displacement under no waves and waves, respectively. The dynamic approach accounts for profile evolution (marked with solid lines). The static approach assumes that vegetation retreats landward under rising sea levels without profile evolution (marked with dashed lines). The comparative analysis is based on the distinct profiles generated after 150 years under varying coastal conditions (Figure 3).

The present study systematically explored mangrove vulnerability under various coastal conditions accounting for dynamic mangrove vegetation and profile evolution. Neglecting such dynamics will cause substantial differences in mangrove vulnerability projections (Figure 11). The application of a simplified approach with static coastal profile and “bed level‐based” vegetation characteristics is only appropriate in small tidal range systems under slow SLR and without wave effects, for which seaward vegetation behaviors are similar to our dynamic approaches (Figure 11a). The contrast between the static and dynamic approach becomes larger if tidal range or sediment supply is increased or additional wind waves are included, and the dynamic approach consistently suggests a lower mangrove vulnerability in comparison to the static approach (Figure 11). This finding not only supports conclusions from previous research that mangrove resilience is enhanced through bio‐physical processes including sediment accretion (Krauss et al., 2014), but also shows that dynamic coastal profiles and vegetation exert distinct controls on mangrove behaviors in the context of SLR between different coastal environments.

### 4.3 Model Limitations and Perspectives

In this study we applied a comprehensive model incorporating detailed descriptions of bio‐morphodynamic processes. While this allows for unraveling the effects of complex bio‐physical interactions, it creates a trade‐off in terms of the spatial scale of model simulations that can be considered. Even though similar types of models have been applied to entire estuarine systems (Brückner et al., 2021), the large number of model simulations carried out here necessitated a one‐dimensional idealized modeling approach. Even though two‐dimensional processes characteristic for estuaries are not incorporated, it is plausible that insights obtained from our idealized profile modeling (e.g., the increased vulnerability of mangroves in micro‐tidal conditions) are applicable to mangroves in estuarine systems as well.

Although our research explores the effects of diverse coastal conditions and accounts for a range of bio‐physical processes, model simulations struggle to capture certain profile characteristics, such as the abrupt slope difference between vegetated and unvegetated sections observed in some mangrove environments (e.g., Figures 1a and 1c). The causal factors can be related to different coastal processes and properties, including (a) variations in sediment composition, grain size and mangrove root density across the profile and through depth resulting in differences in sediment erodibility and potentially causing slope differences (Spenceley, 1977; Swales et al., 2007, 2021); (b) complex tidal forcing including overtides of the M2 tidal constituent, such as the M4 and M6 components which modify tidal asymmetry and influence landward sediment transport and thus profile shape (Wang et al., 1999); (c) energetic wave impacts reinforcing mud erosion at the mangrove forest edge (Winterwerp et al., 2013); (d) variations in mangrove distributions linked to species‐specific colonization requirements such that some mangrove species can only colonize on the elevated, more gentle coastal platform (Chapman, 1976); and (e) 2D effects in the horizontal plane such as tidal creeks and associated morphological features (e.g., Figure 1c) (Boechat Albernaz et al., 2020). Including some of these factors may improve comparisons with real vegetated profiles and potentially influence mangrove behaviors under SLR, but will also raise the difficulty of analyzing the complex bio‐physical interactions controlling mangrove dynamics. The aim of accurately quantifying wetland changes for real mangrove sites can be achieved in the future with extra efforts in parameterizing our model.

Our wave scenarios, adding small waves at the open boundary, should mainly be interpreted as testing the sensitivity of the resulting coastal configuration to an increased resuspension flux potentially caused by wind. Small wind waves with heights between 0.03 and 0.27 m have been observed at various mangrove sites, but particularly develop in shallow water depth environments (Bao, 2011; Brinkman, 2006; Horstman et al., 2014; Mazda et al., 2006; Quartel et al., 2007). The effects of wind waves on morphology have been simulated by a relatively simple model (i.e., “roller model”), with the benefit of high computation efficiency in comparison to more comprehensive wave models such as SWAN (Booij et al., 1999). However, limitations exist as wave attenuation by mangrove vegetation is not included, and thus, spatial variations in sediment deposition and erosion linked to such vegetation‐induced wave attenuation is ignored. In reality, mangroves exert higher drag forces dissipating wave energy and reducing wave height across the flats thereby facilitating sediment settling and contributing to sediment accumulation (Mazda et al., 1997; Quartel et al., 2007). Previous experimental and numerical studies have highlighted that the attenuation of waves by mangroves ranges between 20% and 60% (Phan et al., 2019). Although the omission of wave attenuation by mangroves in this study may overestimate sediment resuspension, wave attenuation depends on wave characteristics with wave attenuation efficiency being typically lower for smaller and shorter waves (Phan et al., 2019), such as in this study. Moreover, wave attenuation by mangroves observed in the field has been shown to vary between tidal systems (Horstman et al., 2014). A gentler coastal slope with shallower water depths is more sensitive to wave dampening by mangroves (Parvathy & Bhaskaran, 2017), implying that wave attenuation effects are more important in micro‐tidal systems. So far, several models have been developed to simulate wave dampening within vegetation but still have some limitations in incorporating morphological changes, especially for long‐term morphodynamic processes (Phan et al., 2019; Wu et al., 2016). Accurately capturing wave attenuation by vegetation and related effects on morphological development should account for the complex interactions between currents and waves over vegetated objects, sediment trapping and entrainment and canopy bending among other factors (Carr et al., 2018; Méndez et al., 1999), so that further research can assess more comprehensively the impacts of waves on mangrove ecosystem vulnerability.

In addition to more complex hydrodynamics, vegetation attributes such as mangrove stem density and the aboveground root system also affect sediment accretion over the vegetated flats (Kumara et al., 2010; Quartel et al., 2007; Xie et al., 2020), which eventually determines the overall functionality of mangrove forests and their capacity to provide coastal protection against the risk of flooding (Gijsman et al., 2021). Previous research has highlighted that dense mangrove vegetation contributes to sediment accretion in the seaward region of the forest, limiting landward sediment accretion and resulting in strong spatial variations in bed level changes, in turn controlling profile shape. However, more sediment can be transported landward under sparse mangrove vegetation, resulting in a more homogeneous accretion over vegetated flats (Xie et al., 2020). Although this study may underestimate the impacts of root density variations, it highlights the role of tidal range and sediment availability on shaping a gentle coastal platform, allowing mangrove colonization and extending mangrove coverage (Figure 3k). Therefore, our bio‐morphodynamics modeling approach, as presented here, may be useful to study the persistence of mangroves for coastal flood protection in the face of SLR impacts.

Finally, the simulations conducted in this study only consider surface accretion, while organic accretion and other sub‐surface processes can also play an important role in controlling surface elevation changes and thus mangrove vulnerability (Breda et al., 2021; Cahoon et al., 2020; Krauss et al., 2014). Organic matter accumulation by production of refractory roots and leaf litter contributes to vertical elevation gain of the soil surface (Middleton & McKee, 2001). Field observations have shown that these organic materials can play an important role in promoting mangrove persistence in the face of SLR, especially in areas with limited sediment inputs (McKee, 2011; McKee et al., 2007). However, other sub‐surface processes, such as land subsidence, sediment compaction and organic decomposition can reduce the surface elevation, even leading to an elevation deficit (Figure S9 in Supporting Information S1). Clearly, the impact of sub‐surface processes on mangrove vulnerability in different coastal conditions needs to be further explored, both through field measurements and modeling, also given the role that these processes play in the sequestration of carbon by mangrove ecosystems (Alongi, 2014).

## 5 Conclusions

Our numerical simulations show that the evolution and characteristics of the vegetated coastal profile, including platform development, is controlled by non‐linear interactions between tidal range, small wind waves and sediment supply. This in turn influences the fate of mangroves under rising sea levels.

Mangrove expansion or retreat due to SLR is a function of tidal range with micro‐tidal systems being the most vulnerable, already retreating under slow SLR rates even with ample sediment supply. In meso‐ and macro‐tidal systems, dynamic profiles driven by sediment accretion across the vegetated flats can protect mangroves from submergence by SLR. For the time period simulated here, mangroves may survive SLR despite limited sediment accretion, as profile change and constraints during vegetation recruitment establish an inundation buffer, and as a consequence, the inundation threshold of mangrove trees is not immediately exceeded during rising sea levels. Still, rising sea levels reduce this inundation buffer such that mangroves are less resilient to ongoing SLR beyond the simulation period. The landward mangrove habitats (i.e., those areas that become suitable for mangrove growth as rising sea levels cause inundation of previously dry land) can be created with limited sediment supply. Nevertheless, infilling of new accommodation space may occur, potentially with sediment originating from erosion and reconfiguration of the lower coastal profile. Although sediment accretion rates across mangrove forests are generally enhanced by SLR rates, the timing and magnitude of accretion vary with coastal conditions and change non‐linearly from the mangrove seaward edge. Overall, our results indicate that environmental conditions drive distinct mangrove responses to SLR and projections of mangrove vulnerability should account for coastal profile adjustments and complex mangrove growth dynamics that emerge from highly non‐linear bio‐morphodynamic feedbacks.

## Conflict of Interest

The authors declare no conflicts of interest relevant to this study.

## Supporting information

Supporting Information S1

## Data Availability

The field data regarding mangrove seaward edge elevation relative to MWL is summarized from previous publications and is available as supplementary materials (Table S4 in Supporting Information S1).The field data regarding local SLR rates and vertical elevation dynamics is available as supplementary material (Table S5 in Supporting Information S1), summarized from the open‐access publication by McKee et al. (2021). Delft3D is an open‐source code available online (at https://oss.deltares.nl). The dynamic vegetation code with a representative model setting is available at https://github.com/xiedanghan/MangroveVulnerabilityModel.
