# Supplementary material for: Implications of Coastal Conditions and Sea‐Level Rise on Mangrove Vulnerability: A Bio‐Morphodynamic Modeling Study
Source: J Geophys Res Earth Surf. 2022 Feb 28;127(3):e2021JF006301. doi: 10.1029/2021JF006301 (PMC9285630; doi:10.1029/2021JF006301)
Supplement: Supplementary file 1 — Supporting Information S1 [file JGRF-127-0-s001.docx]

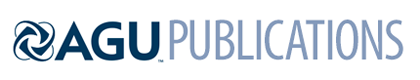


*[Earth Surface]*

Supporting Information for

**Implications of Coastal Conditions and Sea-Level Rise on Mangrove Vulnerability: a Bio-morphodynamic Modelling Study**

Danghan Xie^1^, Christian Schwarz^2,3^, Maarten G. Kleinhans^1^, Zeng Zhou^4^ and Barend van Maanen^5^

^1^Faculty of Geosciences, Utrecht University, Utrecht, Netherlands.

^2^Department of Civil Engineering, Hydraulics and Geotechnics, KU Leuven, Leuven, Belgium.

^3^Department of Earth and Environmental Sciences, KU Leuven, Leuven, Belgium.

^4^State Key Laboratory of Hydrology-Water Resources and Hydraulic Engineering, Hohai University, Nanjing, China.

^5^College of Life and Environmental Sciences, University of Exeter, Exeter, United Kingdom.

**Contents of this file**

Texts S1 to S10

Figures S1 to S10

Tables S1 to S5

**Introduction**

This Supporting Information includes ten texts, ten supplementary figures (Figures S1 to S10), five tables (Tables S1 to S5). Texts S1 to S10 will serve as further explanation of Figures S1 to S10.

Figure S1 shows the sensitivity analysis of drag coefficients in influencing the model results. Figure S2 displays the shape of two key factors controlling mangrove growth rate based on equations 5-6. Figure S3 shows the profile evolution with different combinations of tidal range and initial coastal slopes. Figure S4 is the zoomed in version of Fig. 5 in the main text. Figures S5 to S10 are model results supporting the main findings of this research. Figure S5 shows the spatio-temporal changes in the net sediment transport rate in the first 50 years. Figure S6 shows the sediment transport rate during flood and ebb at 3 particular years. Figure S7 displays the relations between mangrove seaward edge elevation and tidal ranges. Figure S8 exhibits the tidal prism throughout 250 years, including the first 150 years without sea-level rise followed by 100 years with sea-level rise. Figure S9 shows the vertical changes in numerical simulations and field observations. Figure S10 provides information on the changes in relative hydroperiod, suspended sediment concentration and bed shear stress at mangrove seaward edge over 250 years.

Tables S1 and S2 show the parameter settings of the hydro-morphodynamic model and the vegetation model, respectively. Table S3 displays the overview of model simulations conducted with different coastal conditions, including tides, waves, sediment supply and sea-level rise scenarios. Table S4 provides data showing mangrove seaward edge elevation above MWL at different field sites. Table S5 presents the field observation data regarding sea-level rise rates and surface elevation change, surface accretions rates and shallow subsidence at several different mangrove sites, summarized by McKee et al. (2021).

Text S1.

Figure S1 shows how the model results change with different drag coefficients (stems and roots), *C_D_*. The panels display comparisons of bed level, 90th percentile depth-averaged velocity and suspended sediment concentration (SSC) across the coastal profiles in year 250. For lower drag coefficients (i.e. 0.5 for stems and 0.1 for roots) there will be a higher platform landward, with slightly larger velocities but lower suspended sediment concentrations in the intertidal area, causing the seaward vegetation edge to be positioned more landward. However, other combinations with larger drag coefficients show similar outcomes, with comparable velocity and sediment distributions. The seaward vegetation edge remains at the same cross-shore location; however, the seaward vegetation edge will occur at a slightly higher bed elevation with smaller drag (i.e. 1.5 for stems and 1 for roots). Thus, our chosen drag coefficients cover the characteristic behavior which is representative of a wide range of previously reported drag coefficients (i.e. 0.7-3.5; based on Nepf (1999) and Horstman et al. (2018).


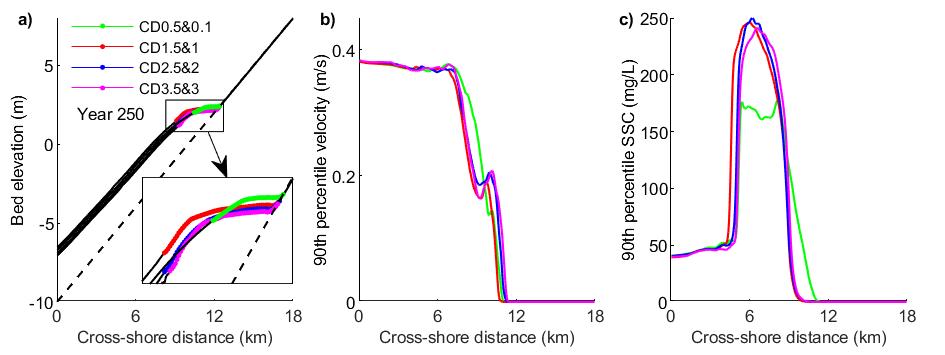


**Figure S1.** Comparisons of bed level, 90^th^ percentile of depth-averaged velocity and suspended sediment concentration (SSC) across the coastal profiles with different *C_D_* settings. The comparisons are developed under similar coastal conditions, that is, with large tidal range (5 m) and intermediate sediment supply (30 mg/L) without wave effects in the year 250. Green, red, blue and purple represent different *C_D_* combinations and the drag coefficients for stems and roots are marked in the legend in subplot a). Green, red, blue and purple color dots in a) indicate mangrove presence along their corresponding profiles, which are shown as solid lines. The dashed lines indicate the initial bed profile. In this research, we use *C_D_* = 1.5 for stems and *C_D_* = 1.0 for roots.

Text S2.

Figure S2 shows how the inundations stress factor changes with relative hydroperiod and how the competition stress factor changes with existing biomass within one grid cell. In the vegetation model, the product of these two factors determines the growth rates of mangrove trees and also serves as a key parameter evaluating 1) colonization conditions and 2) mortality conditions.


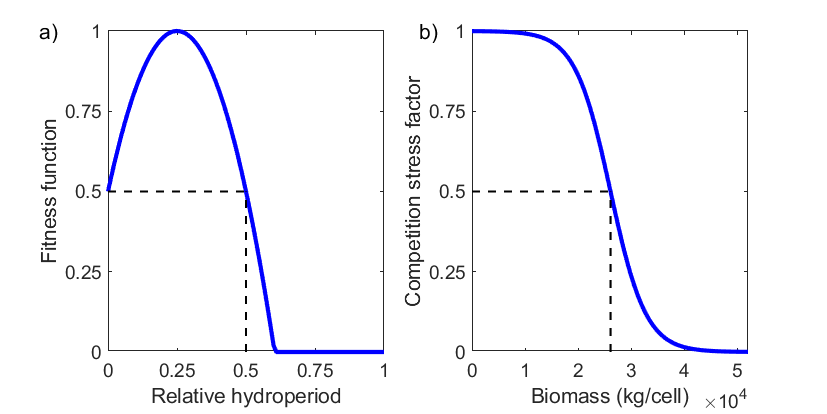


Figure S2. Growth control factors used in the vegetation model (van Maanen et al., 2015; Xie et al., 2020). (a) Fitness function, characterized by an optimal relative hydroperiod (P = 0.25). P > 0.6 implies over-inundation so mangroves stop growing completely. (b) Competition stress factor, representing the competition between trees as neighbouring trees have to share resources.

Text S3.

Figure S3 shows how the profile shapes evolve under different initial coastal slopes forced by three tidal ranges. Profiles with a gentle slope in micro-tidal range systems, with a medium slope in meso-tidal range system and with a steep slope in macro-tidal range system, show relatively small adjustment before a stable configuration is reached. Other combinations will cause significant deposition or erosion along the profile before the stable profile is achieved.


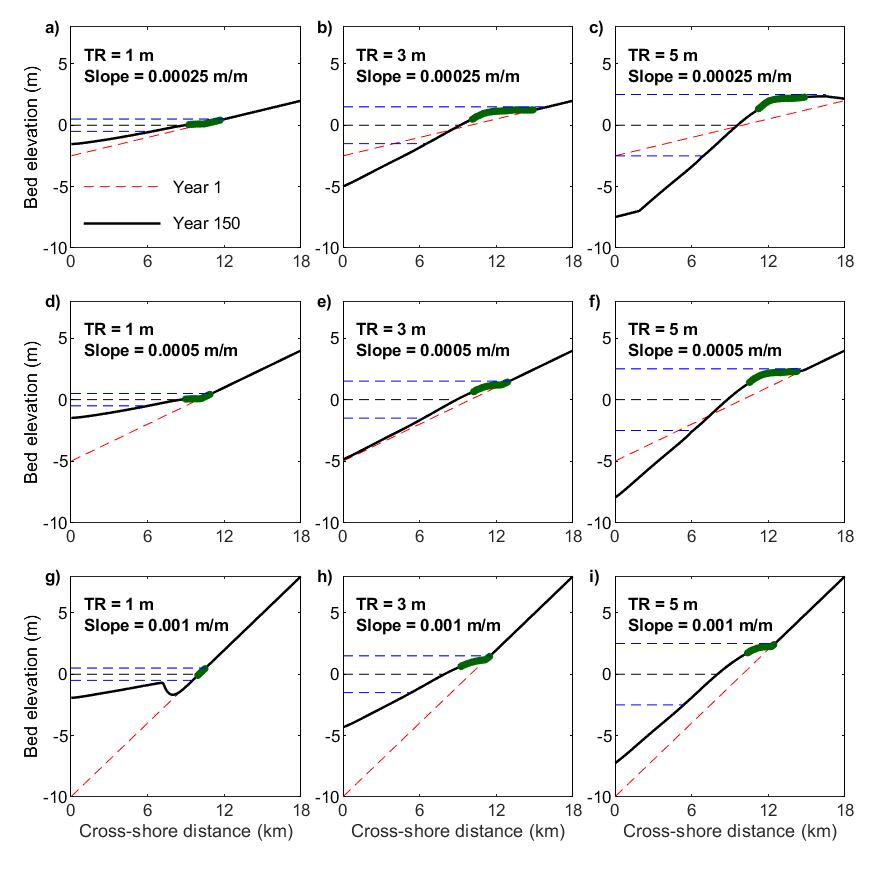


Figure S3. Bed level evolution under different combinations of tidal ranges and initial coastal slopes. The comparisons are based on intermediate sediment supply (30 mg/L) without wave effects. Bed level initialized with a-c) gentle coastal slope, d-f) medium coastal slope and g-i) steep coastal slope. a,d&g), b,e&h) and c,f&i) indicate the micro-, meso- and macro-tidal range systems, respectively. Red dashed lines show the initial bed level, while black solid lines with green dots show the bed level with the location of mangroves in year 150. TR and Slope represent the tidal range and initial coastal slope applied in the model.

Text S4.

Figure S4 is a zoomed in version of Figure 5 to highlight differences between scenarios.


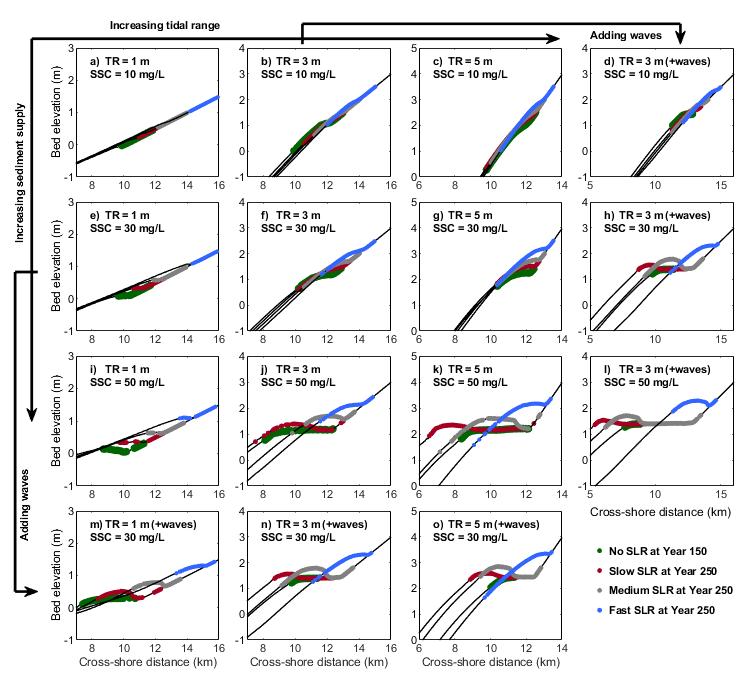


Figure S4. Zoomed in version of Figure 5 in the main text showing mangrove behaviors in response to different rates of SLR.

Text S5.

Figure S5 shows how the sediment transport rate changes during the first 50 years in the absence and presence of waves. For similar tidal range systems, models with and without waves are initialized with the same bathymetry. Profile accumulation during the first 50 years is facilitated due to the net sediment flux in the flood direction (Fig. S5). The onshore net sediment flux is further enhanced by wave effects, especially in the micro- and meso-tidal systems where water depths are shallower (Fig. S5d-e).


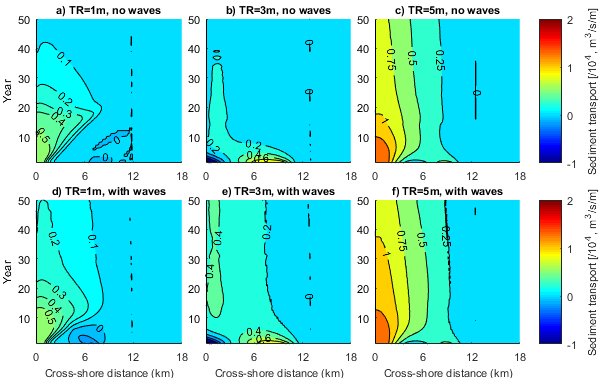


Figure S5. Spatio-temporal distribution of sediment transport rate under intermediate sediment supply (30 mg/L) over the first 50 years, without (a, b, c) and with (d, e, f) wave effects. Results are presented for micro-tidal (a, d), meso-tidal (b, e) and macro-tidal (c, f) conditions. The sediment transport rate is calculated over an entire tidal cycle.

Text S6.

Figure S6 shows the difference in sediment flux during flood and ebb every 50 years. In general, small wind waves enhance sediment flux during the flood period more than during the ebb period, causing a larger net sediment input in the flood direction.


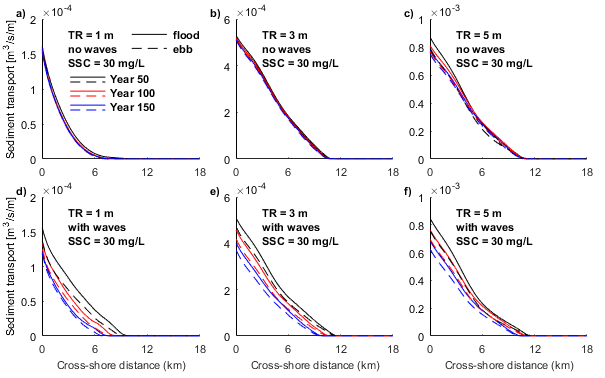


Figure S6. Sediment fluxes under intermediate sediment supply (30 mg/L) during the flood (solid line) and ebb periods (dashed line) evaluated over an entire tidal cycle in three particular years (50, 100 and 150), without and with wave effects. Results are presented for micro-tidal (a, d), meso-tidal (b, e) and macro-tidal (c, f) conditions. TR and SSC represent tidal range and suspended sediment concentration, respectively.

Test S7.

Figure S7 compares the observed mangrove elevation with our model results for different tidal ranges. Both the model and field data show that an increased tidal range limits mangrove seaward colonization and constrains mangroves to higher bed elevations.


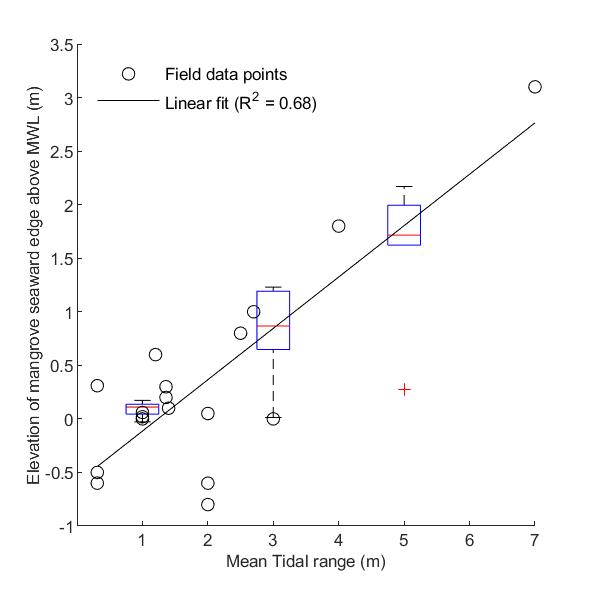


Figure S7. Comparing the location of the mangrove seaward edge relative to MWL between modelling results (box plots) and field observations (Table S4). Each box plot is based on 6 simulated mangrove seaward edge elevations at year 150, accounting for the combinations of 3 sediment supply settings (10, 30 and 50 mg/L) and 2 wave conditions (no waves and with waves).

Text S8.

Figure S8 shows that the tidal prism first decreases as profile accumulates before sea-level rise, and then increases when sea-level rise occurs which creates new landward accommodation space.


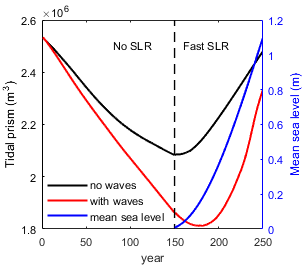


Figure S8. Temporal change of tidal prism under intermediate sediment supply (30 mg/L) and large tidal range (5m) throughout 250 years, with and without wave effects. The first 150 years were simulated without sea-level rise, followed by a 100-year fast-sea level rise. Black and red lines represent the changes of tidal prism without and with waves, respectively. The fast sea-level rise curve is shown in blue.

Test S9.

Figure S9 shows sediment accretion calculated through our numerical modelling is comparable to field observations, which shows that our model is able to capture above-ground sedimentary processes throughout different coastal environmental conditions. In the meanwhile, that organic accretion can create an elevation gain that is of a similar magnitude as inorganic accretion, which however will be offset by other sub-surface processes potentially resulting in a net elevation deficit. Field data in Fig. S9c is based on previous studies (McKee, 2011; McKee et al., 2021).


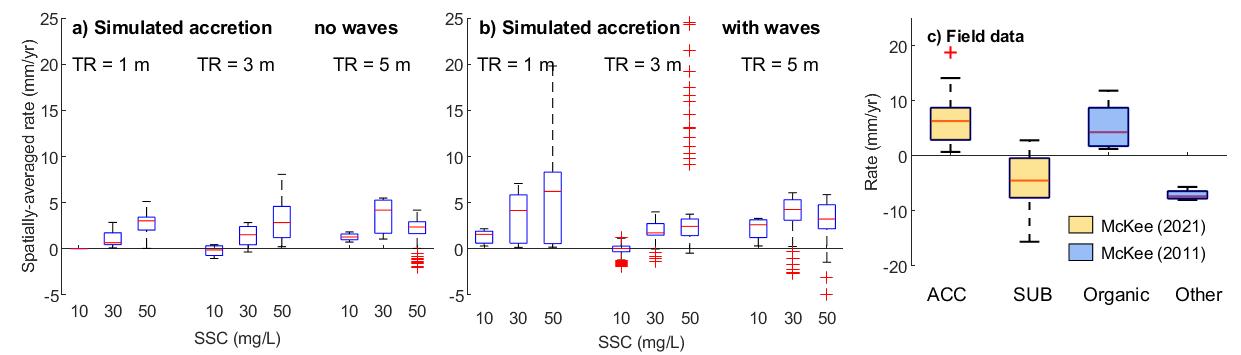


**Figure S9**. Box plots of vertical changes in numerical simulations and field observations. Spatially-averaged sediment accretion rates across different simulated scenarios without waves (a) and with waves (b); field observations on elevation change due to sediment accretion (ACC) and sub-surface processes (SUB) at different mangrove sites (yellow) (c). The sub-surface processes are further divided into organic matter accretion (Organic) and other sub-surface processes (Other), such as land subsidence, sediment compaction and organic decomposition (blue). The rates in a) and b) are averaged over the vegetated areas and computed for a 100-year time period.

Text S10.

Figure S10 shows the temporal changes of relative hydroperiod, 90^th^ percentile of suspended sediment concentration and bed shear stress at one fixed cross-shore location. In this analysis, the fixed location is based on mangrove seaward edge in year 150, which is the seaward green dots in each subplot of Figure 5 (without waves). Before sea-level rise, the relative hydroperiod generally decreases over years. Sediment concentration and bed shear stress remain stable in micro-tidal systems but drop over time in meso- and macro-tidal range systems. When sea level starts to increase, although the bed shear stress increases, the relative hydroperiod and sediment concentration at the front of mangrove forests also increase, leading to enhancing sediment accretion rates.


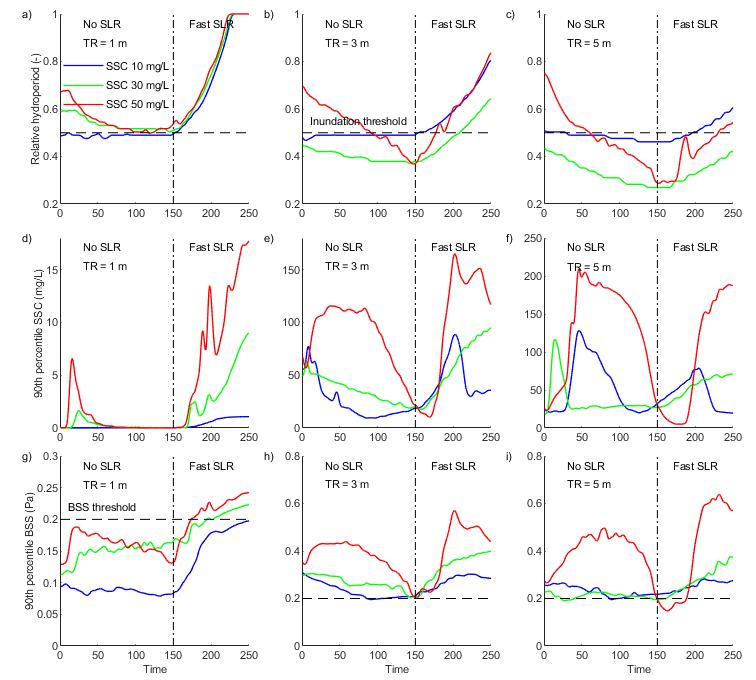


Figure S10. Temporal changes in relative hydroperiod, 90^th^ percentile of suspended sediment concentration (SSC) and 90th percentile bed shear stress (BSS) at the mangrove seaward edge over 250 years (without waves). The first 150 years were simulated without sea-level rise, followed by a 100-year fast-sea level rise.

| **Category** | **Applicable situation** | **Parameter** | **Value/Description** | **Unit** |
| --- | --- | --- | --- | --- |
| **Time reference** | Not required | Hydrodynamic time step | 0.5 | min |
|  | Not required | Morphological acceleration factor | 30 | - |
|  | Not required | Ecological simulation period | 250 | years |
| **Domain** | Not required | Grid size | 50 × 50 | m |
|  | Micro-tidal system slope | Slope | 0.00025 | m/m |
|  |  | Domain size (x × y) | 18000 × 4.5 (-2.5 to 2) | m |
|  | Meso-tidal system slope | Slope | 0.0005 | m/m |
|  |  | Domain size (x × y) | 18000 × 9 (-5 to 4) | m |
|  | Macro-tidal system slope | Slope | 0.001 | m/m |
|  |  | Domain size (x × y) | 18000 × 18 (-10 to 8) | m |
| **Boundary condition** | Wave | Significant wave height | 5 | cm |
|  |  | Peak wave period | 1 | s |
|  | Micro-M2 tide | Tidal range | 1 | m |
|  | Meso-M2 tide | Tidal range | 3 | m |
|  | Macro-M2 tide | Tidal range | 5 | m |
| **Sediment** | Not required | Critical bed shear stress for erosion | 0.2 | N/m^2^ |
|  | Not required | Critical bed shear stress for deposition | 1000 | N/m^2^ |
|  | Not required | Settling velocity | 5× 10^-4^ | m/s |
|  | Not required | Erosion parameter | 5× 10^-5^ | kg/m^2^/s |

Table S1. Hydro-morphodynamic model parameter settings

| **Category** | **Parameter** | **Value/Description** | **Unit** |
| --- | --- | --- | --- |
| **Vegetation parameters** | Initial stem diameter, *D_0_* | 1.37 | cm |
|  | Maximum root number, *N_roots,max_* | 1000 | - |
|  | Root diameter, *D_roots_* | 1 | cm |
|  | Root height, *H_roots_* | 15 | cm |
|  | Drag coefficient of roots, *C_Dr_* | 1 | - |
|  | Drag coefficient of stems, *C_Ds_* | 1.5 | - |
| **Growth parameters** | Maximum stem diameter | 40 | cm |
|  | Maximum tree height | 1000 | cm |
|  | Growth constant, *G* | 12.68 | cm/month |
|  | Growth constant, *b_2_* | 43 | - |
|  | Growth constant, *b_3_* | 0.536 | cm^-1^ |
|  | Fitness function constant, *a* | -8 | - |
|  | Fitness function constant, *b* | 4 | - |
|  | Fitness function constant, *c* | 0.5 | - |
|  | Competition stress factor constant, *d* | -0.0003 | - |
|  | Roots formula constant, *k* | 0.3 | - |

Table S2. Dynamic vegetation model parameter settings.

| **Tidal systems** | **Tidal range (m)** | **Sediment supply (mg/L)** | **Wave height (cm)** | **Sea-level rise (SLR)** |
| --- | --- | --- | --- | --- |
| **Micro** | 1 | 10, 30 or 50 | 0 or 5 | no SLR, RCP2.6, RCP4.6 or RCP8.5 |
| **Meso** | 3 |  |  |  |
| **Macro** | 5 |  |  |  |

Table S3. Overview of coastal conditions explored in our study, amounting to 72 runs in total covering all combinations.

| **ID** | **Location** | **TR (m)** | **Elevation above MWL (m)** | **Source** |
| --- | --- | --- | --- | --- |
| 1 | Dongzhaigang Bay, China | 1 | 2.00E-02 | Fu et al. (2019) |
| 2 |  |  | 0 |  |
| 3 |  |  | 6.00E-02 |  |
| 4 | Futian, China | 1.36 | 0.2 | Chen et al. (2021) |
| 5 |  |  | 0.3 |  |
| 6 | Macouria bank, French Guiana | 2 | 5.00E-02 | Anthony et al. (2008) |
| 7 | Sinú River Delta, Colombian Caribbean | 0.31 | -0.5 | Sánchez-Núñez et al. (2019) |
| 8 |  |  | 0.31 |  |
| 9 |  |  | -0.6 |  |
| 10 | The Firth of Thames, New Zealand | 2.7 | 1 | Swales et al. (2015) |
| 11 | Mekong delta, Vietnam | 4 | 1.8 | Phan et al. (2015) |
| 12 | Cù Lao Dung, Vietnam | 1.4 | 0.1 | Bryan et al. (2017) |
| 13 | Queensland, Australia | 1.2* | 0.6 | Knight et al. (2009) |
| 14 | Kantang, Thailand | 2 | -0.6 | Horstman et al. (2014) |
| 15 | Palian, Thailand |  | -0.8 |  |
| 16 | Mahé Island, Seychelles | 3 | 0 | Sefton and Woodroffe (2021) |
| 17 | Sundarbans, Bangladesh | 7 | 3.1 | Ellison et al. (2000) |
| 18 | Matapouri Estuary, New Zealand | 2.5 | 0.8 | Andrea (2006) |

(*) Tidal range (TR) is calculated from TPXO tide models (https://www.tpxo.net/home).

**Table S4.** Overview of field observations showing tidal ranges and mangrove seaward edge elevations relative to mean water level (MWL).

| **ID** | **Location** | **Site** | **Surface elevation change (mm/yr)** | **Surface accretion (mm/yr)** | **RSLR (mm/yr)** | **Shallow subsidence /expansion (mm/yr)** | **Source** |
| --- | --- | --- | --- | --- | --- | --- | --- |
| 1 | Rookery Bay, Florida, USA | Fringe | 3.5 | 7.8 | 6.6 | -4.3 | Cahoon and Lynch (1997) |
| 2 |  | Basin | 3.7 | 6 | 4.6 | -2.3 |  |
| 3 |  | Island shoreline | 2.5 | 6.3 | 6.1 | -3.8 |  |
| 4 |  | Island interior | 0.6 | 4.4 | 6.1 | -3.8 |  |
| 5 | Gulf of Fonseca, Honduras | Interior | 4.6 | 2.9 | -0.7 | 1.7 | Cahoon et al. (2002) |
| 6 |  | Shoreline | 4.6 | 2.9 | -0.7 | 1.7 |  |
| 7 | Roatan, Honduras | Interior | 4.8 | 2 | -0.8 | 2.8 | Cahoon et al. (2003) |
| 8 |  | Shoreline 1 | 4.8 | 2 | -0.8 | 2.8 |  |
| 9 |  | Shoreline 2 | 4.8 | 2 | -0.8 | 2.8 |  |
| 10 | New South Wales, Australia | Cararma Inlet | -0.8 | 3 | 8 | -3.8 | Rogers et al. (2006) |
| 11 |  | Currambene Creek | 0.3 | 0.7 | 4.5 | -0.4 |  |
| 12 |  | Homebush Bay | 5.6 | 4.6 | -0.2 | 1 |  |
| 13 |  | Kooragang Island | 1.9 | 4.7 | 3.1 | -2.8 |  |
| 14 |  | Ukerebagh Island | 2.2 | 2.2 | -0.4 | 0 |  |
| 15 |  | Minnamurra River | 0.6 | 6.6 | 5.6 | -6 |  |
| 16 | Victoria, Australia | French Island | -2.1 | 9.5 | 14.3 | -11.6 |  |
| 17 |  | Kooweerup | 0 | 7.2 | 9.9 | -7.2 |  |
| 18 |  | Quail Island | -2.6 | 6.8 | 12 | -9.4 |  |
| 19 |  | Rhyll/Phillip Island | 0.9 | 5.1 | 6.8 | -4.2 |  |
| 20 | Twin Cays, Belize | Transition | -1.1 | 2 | 5.4 | -3.1 | McKee et al. (2007) |
| 21 |  | Dwarf | -3.7 | 0.7 | 6.7 | -4.4 |  |
| 22 |  | Fringe | 4.1 | 1.6 | -0.2 | 2.5 |  |
| 23 | Kosrae, Micronesia | Yela shoreline | -3 | 11.6 | 16.4 | -14.6 | Krauss et al. (2010) |
| 24 |  | Yela riverine | -2.7 | 12.9 | 17.4 | -15.6 |  |
| 25 |  | Yela interior | 1.3 | 12 | 12.5 | -10.7 |  |
| 26 |  | Utwe shoreline | 1.2 | 11.9 | 12.5 | -10.7 |  |
| 27 |  | Utwe riverine | 6.3 | 18.7 | 14.2 | -12.4 |  |
| 28 |  | Utwe interior | 1.3 | 12.9 | 13.4 | -11.6 |  |
| 29 | Pohnpei, Micronesia | Sapwalap riverine | -0.6 | 14.1 | 16.5 | -14.7 |  |
| 30 |  | Sapwalap shoreline | -2.3 | 4.1 | 8.2 | -6.4 |  |
| 31 |  | Sapwalap interior | 0.9 | 8.2 | 9.1 | -7.3 |  |
| 32 |  | Enipoas shoreline | -5.8 | 6.6 | 14.2 | -12.4 |  |
| 33 |  | Enipoas riverine | -1.4 | 6.3 | 9.5 | -7.7 |  |
| 34 |  | Enipoas interior | -2.8 | 2.9 | 7.5 | -5.7 |  |

*(Continued)*

| **ID** | **Location** | **Site** | **Surface elevation change (mm/yr)** | **Surface accretion (mm/yr)** | **RSLR (mm/yr)** | **Shallow subsidence**  **/expansion (mm/yr)** | **Source** |
| --- | --- | --- | --- | --- | --- | --- | --- |
| 35 | Everglades, Florida, USA | Shark River | 1.4 | 6.5 | 7 | -5.1 | Whelan et al. (2009) |
| 36 | Rookery Bay, Florida, USA | Fringe | 0.6 | 5.7 | 7.2 | -5.1 | McKee (2011) |
| 37 |  | Basin 1 | 3.9 | 2 | 0.2 | 1.9 |  |
| 38 |  | Basin 2 | 1.1 | 7.6 | 8.6 | -6.5 |  |
| 39 | Queensland, Australia | Moreton Bay east | 5.8 | 8 | 4.1 | -2.2 | Lovelock et al. (2015) |
| 40 |  | Moreton Bay west | 1.7 | 9.2 | 9.4 | -7.5 |  |
| 41 | Louisiana, USA | Port Fourchon | 3.8 | 9.8 | 15 | -6 | McKee and Vervaeke (2018) |
| 42 | Hainan, China | Sanjiang | 8.6 | 13.2 | 10.9 | -4.6 | Fu et al. (2018) |
| 43 |  | Houpai | 2.2 | 2.7 | 6.8 | -0.5 |  |
| 44 |  | Houpai | 7.8 | 7.4 | 5.9 | 0.4 |  |

Table S5. Summary of field data on vertical elevation dynamics across different mangrove hydrogeomorphic settings, including surface elevation change (mm/yr), surface accretion, relative sea-level rise (mm/yr) and shallow subsidence (mm/yr). The shallow subsidence (negative value) or expansion (positive value) is calculated from the differences between surface elevation change and surface accretion. Data was summarized by McKee et al. (2021).
